# Microfluidic Manufacture of Lipid-Based Nanomedicines

**DOI:** 10.3390/pharmaceutics14091940

**Published:** 2022-09-14

**Authors:** Karim Osouli-Bostanabad, Sara Puliga, Dolores R. Serrano, Andrea Bucchi, Gavin Halbert, Aikaterini Lalatsa

**Affiliations:** 1 Biomaterials, Bio-Engineering and Nanomedicine (BioN) Lab, Institute of Biomedical and Biomolecular Sciences, School of Pharmacy and Biomedical Sciences, University of Portsmouth, White Swan Road, Portsmouth PO1 2DT, UK; 2 School of Pharmacy and Biomedical Sciences, Robertson Wing, University of Strathclyde, 161, Cathedral Street, Glasgow G4 0RE, UK; 3 Pharmaceutics and Food Technology Department, School of Pharmacy, Universidad Complutense de Madrid, Plaza Ramón y Cajal s/n, 28040 Madrid, Spain; 4 Facultad de Farmacia, Instituto Universitario de Farmacia Industrial, Universidad Complutense de Madrid, 28040 Madrid, Spain; 5 School of Mechanical and Design Engineering, Faculty of Technology, University of Portsmouth, Portsmouth PO1 3DJ, UK; 6 CRUK Formulation Unit, School of Pharmacy and Biomedical Sciences, Robertson Wing, University of Strathclyde, 161, Cathedral Street, Glasgow G4 0RE, UK

**Keywords:** nanomedicine, microfluidics, liposomes, manufacture, engineering, scale-up

## Abstract

Nanoparticulate technologies have revolutionized drug delivery allowing for passive and active targeting, altered biodistribution, controlled drug release (temporospatial or triggered), enhanced stability, improved solubilization capacity, and a reduction in dose and adverse effects. However, their manufacture remains immature, and challenges exist on an industrial scale due to high batch-to-batch variability hindering their clinical translation. Lipid-based nanomedicines remain the most widely approved nanomedicines, and their current manufacturing methods remain discontinuous and face several problems such as high batch-to-batch variability affecting the critical quality attributes (CQAs) of the product, laborious multistep processes, need for an expert workforce, and not being easily amenable to industrial scale-up involving typically a complex process control. Several techniques have emerged in recent years for nanomedicine manufacture, but a paradigm shift occurred when microfluidic strategies able to mix fluids in channels with dimensions of tens of micrometers and small volumes of liquid reagents in a highly controlled manner to form nanoparticles with tunable and reproducible structure were employed. In this review, we summarize the recent advancements in the manufacturing of lipid-based nanomedicines using microfluidics with particular emphasis on the parameters that govern the control of CQAs of final nanomedicines. The impact of microfluidic environments on formation dynamics of nanomaterials, and the application of microdevices as platforms for nanomaterial screening are also discussed.

## 1. The Nanomedicine Market and Bottlenecks to Market Entry

Nanomedicine is the application of nanotechnology in the medical field with important advances in terms of drug delivery, in vitro and in vivo diagnostics and imaging, regenerative medicine, and local implanted devices [1,2]. Nanoparticulate technologies have revolutionized drug delivery, allowing for passive and active targeting, altered biodistribution, controlled drug release (temporospatial or triggered), enhanced stability, improved solubilization capacity, and a reduction in dose and adverse effects. Nanomedicines can employ hard (inorganic) or soft nanomaterials and are disease-centered, while they combine a molecular understanding of cellular processes with capabilities to produce nanoscale material in a controlled manner for the diagnosis and treatment of diseases [3]. Nanopharmaceuticals can be developed either as drug delivery systems of biologically active drug products consisting of at least two components, one of which is the active ingredient [4].

The nanomedicine market is currently worth more than 150 billion USD, and this value is expected to rise to 334 billion USD by 2025 [5,6]. The market has considerably expanded in recent years due to numerous applications for the treatment of cancer, pain, and infections, as well as due to advances in drug delivery, and the increasing global incidence of cancer is estimated to be a key factor influencing industry growth. More than 50 nanomedicines have been clinically approved [3,4,7,8,9,10,11,12,13,14,15,16,17,18,19] after the initial approval of liposomal doxorubicin (Doxil^®^) for myeloma (multiple myeloma) due to the reduced cardiotoxicity of this formulation compared to unentrapped doxorubicin hydrochloride aqueous solutions [3], while more than 15 are in clinical trials and 75 are in the preclinical phase[9] Additionally, new applications in vaccinations as demonstrated by the formulation of mRNA vaccines in the recent COVID-19 pandemic are also currently contributing to the growth of the market [1,20]. Although data on the use of nanomedicines indicate that, in recent years, applications of nanomedicines have achieved considerable success, time their commercialization simultaneously suffers from many challenges and obstacles [21,22].

The current regulatory framework of the European Medicine Agency (EMA) focuses on the risk/benefit ratio, requiring that nanomedicines are subjected to toxicology and ecotoxicology studies, as well as remain under pharmacovigilance once marketed [23]. The Food and Drug Administration (FDA) has no specific regulatory framework for nanomedicines, but has recently published draft guidance for industry and special guidance for liposomal nanomedicines that are leading the entry into the market [24]. Although the FDA does not clearly separate biological products on the nanometer scale from nanoparticles, when considering whether a product involves the application of nanotechnology, it assesses whether a material or end product is engineered to exhibit properties or phenomena (physical, chemical, or biological) that are attributable to its dimensions, even if one of these dimensions falls outside the nanoscale range and is up to 1 μm [1,3]. 

The major bottlenecks in the uptake of nano-enabling technologies in the market involve difficulties in achieving relevant physiological test results in conventional pre-screening platforms (in vitro), technical issues, including reliance on batch manufacturing to control of manufacturing qualities, the lack of a clear legislative framework, and economic risks as R&D is carried out mainly by small and medium-sized enterprises as big industries do not want to take risk on projects that have not yet been validated [25,26]. Once their potential and feasibility are demonstrated, big pharma is likely to buy the small–medium enterprises (SMEs) or license the products. Thus, to facilitate their technology readiness and scale-up to human studies, successful fabrication of nanomedicines with processes that can be continuous and able to match high-quality standards under GMP is critical. 

## 2. Lipid-Based Nanomedicines

**Figure 1:**
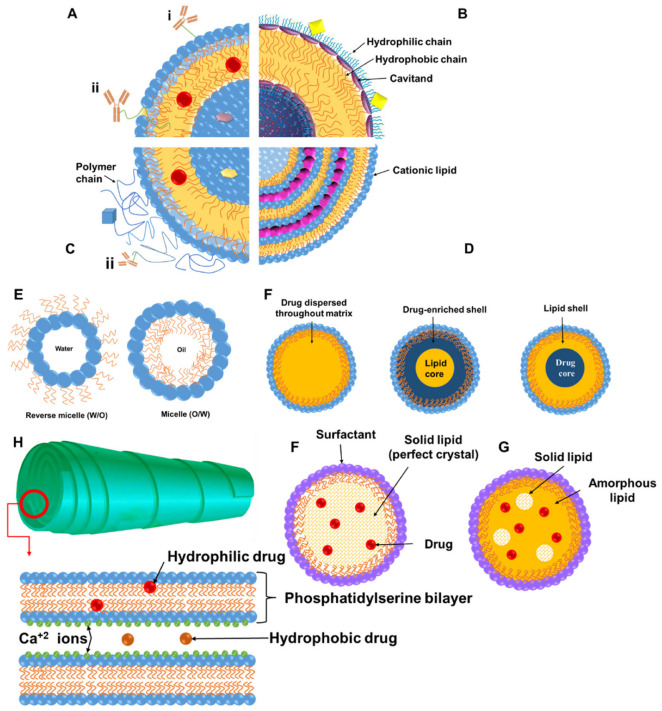
**Schematic representation of lipid-based nanomedicines. Liposomes** (**A**–**D**): Hydrophobic molecules up to few nm in diameter can be entrapped in the phospholipid bilayer (red spheres), while hydrophilic cargo can be loaded in the core (purple pentagon) and their surface can be modified antibodies (hydrophobically anchored (i) or conjugated via a linker or a hydrophilic polymer chain (immunoliposomes (ii)) (**A**). Liposomes with cavitands able to allow host–guest chemical reactions with molecules of complementary shape or size to allow loading in the bilayer, cavitands, and core (**B**). Stealth liposomes and targeted stealth liposomes where the liposome surface is decorated with hydrophilic polymer chains such as polyethylene glycol or a stimulus-responsive polymer, and a targeting moiety or diagnostic moiety (blue square) can be conjugated (peptides, cell-penetrating peptides, and antibodies). Drugs, genetic material, or diagnostic agents (gold, silver, or magnetic particles) can be loaded in the bilayer, core, or surface via conjugation, and lipids can be negatively or positively charged (preferred for complexation with DNA/RNA). **Micelles or inverse micelles** (**E**) are prepared via self-assembly of amphiphiles such as phospholipids and can load hydrophobic or hydrophilic molecules. **Solid lipid nanoparticles (SLNs)** (**F**) are colloidal carriers where liquid lipids have been substituted by a solid lipid, offering unique properties such as small size, large surface area, high drug loading, and the interaction of phases at the interfaces, and they are attractive for their potential to improve performance of pharmaceuticals, nutraceuticals, and other materials, appearing in three forms depending on where drug is loaded (homogeneous matrix (melting point of drug equal to that of lipid), lipid-enriched core (melting point of drug < lipid), and drug-enriched core (melting point of drug > lipid)). **Nanostructured lipid carriers (NLCs)** (**G**) are colloidal carriers prepared by blending of solid lipids with oils, but the matrix remains solid at body temperature to overcome problems of SLNs (low payload for drugs, drug expulsion during storage, and high water content of SLN dispersions). **Cochleates** (**H**) are phospholipid–calcium precipitates derived from the interaction of anionic lipid vesicles with divalent cations such as calcium with a multilayered structure consisting of large and continuous lipid bilayer sheets rolled up in a spiral structure with no internal aqueous phases.

Lipid-based nanomedicines are prepared by bottom-up self-assembly methodologies and can be divided into the following broad categories on the basis of their physicochemical characteristics and fabrication methods such as liposomes, micelles, transferosomes, ethosomes, solid lipid nanoparticles, cochleates, and nanostructured lipid carriers (Figure 1, Table 1) [27], although others exist. Most of these nanoparticulate carriers result in spherical particles that possess at least one internal aqueous compartment surrounded by a single or double lipid layer and offer advantages in terms of high bioavailability, biocompatibility, drug loading, and permeability enhancement [28,29]. Most of the FDA-approved nanomedicines fall under this class of lipid-based nanomedicines [9,30]. 

Liposomes are the most widely approved lipid-based medicines and are typically prepared using phospholipids and cholesterol in multi- or unilamellar vesicles able to entrap lipophilic drugs in the bilayer and hydrophilic drugs in the aqueous internal compartment [31]. Their in vitro and in vivo stability, efficacy, and toxicity can be tuned by manipulating their surface charge, size, lipid composition, number of lamellas in the vesicles, and surface decoration with polymers such as polyethylene glycol or ligands, which allows for a versatile carrier for a range of clinical applications for passively or actively targeted strategies [29,32]. As the reticuloendothelial system can promptly take up liposomes, they often have surface modifications with polymers to improve their circulation half-life on the basis of the clinical application intended [28,33]. 

Transferosomes are lipid-based vesicular carriers that, compared to the rigid lipid bilayers (liposomes) or nonionic surfactant single layer vesicles (niosomes), are elastic, ultra-deformable, and stress-responsive [34]. Transferosomes are composed by four key elements: (i) phospholipids (such as phosphatidylcholine, dipalmitylphosphatidylcholine, distearylphosphatidylcholine), (ii) an edge activator such as a surfactant or bile salt ranging from 10% to 25% (e.g., sodium cholate, sodium deoxycholate, Tween^®^ 80, Span^®^ 80, and dipotassium glycyrrhizinate) [35], (iii) ethanol in a lower percentage usually below 10% (as higher concentrations are described as ethosomes), and (iv) water as a vehicle. In addition to phospholipids, they contain cholesterol or another edge activator such as bile salts and, in some cases, a small quantity of ethanol, typically below 10% [34,36]. The word transferosome is a registered trademark by the German company IDEA AG and the name derives from the Latin word “transferre” meaning “to carry across” and the Greek word “soma” meaning “body”. The technology was first described in 1991 by Çevc and Blume and has been the subject of several patents and research over the last 30 years [34]. Transferosomes are highly ultra-deformable and are able to squeeze through biological barriers such as the stratum corneum (SC) and penetrate as intact vesicles through the skin when their size is below 300 nm and when they are applied under nonocclusive conditions, which maintains the trans-epidermal osmotic gradient that acts as the driving force for the elastic transport into the skin [35,37,38]. The edge activator plays a key role as it provides a high radius of curvature that can destabilize the lipid bilayer, increasing the deformability of the membrane. This allows transferosomes to spontaneously squeeze though channels in the SC that are less than one-tenth the diameter of the vesicles, preventing vesicle rupture when crossing through the different skin layers [37,38]. The concentration of the edge activator in the formulation (usually between 10% and 20%) is crucial and ideally included in sublytic concentrations, i.e., not able to cause destruction of vesicles [35,37,39]. The risk of formation of mixed micelles increases when amounts of edge activator greater than 15% are used [40].

Ethosomes are phospholipid bilayer particles that incorporate alcohols (<10%) to impart a high degree of flexibility to the vesicle membranes, allowing relatively large vesicles to traverse the small intercellular pores within the SC. Ethosomes are soft, malleable vesicles that can range between 30 nm and several microns. Their size is smaller than that of liposomes prepared under the same conditions without the need of a size reduction step due to the high concentration of ethanol employed (20–45% typically) [41,42]. Additionally, ethanol confers a strong negative charge on the vesicles [43]. However, for systemic delivery through the bloodstream, both transferosomes and ethosomes are not ideal since large and flexible lipid-based particles are subject to rapid opsonization and phagocytotic clearance.

Bilosomes, similar to transferosomes, but without incorporating the alcohol content, are bile-salt-stabilized vesicles (bilayers) that have been applied in the oral delivery of antigens, proteins, and peptides [44]. Inclusion of bile salts into the lipid bilayers makes them repulsive to the intestinal bile salts in the gastrointestinal tract and, thus, offer great oral stability [45]. Additionally, these bile salts such as sodium glycocholate (SGC), sodium deoxycholate (SDC), and sodium taurocholate (STC) are also used as intestinal penetration enhancers as they enhance the low aqueous solubility of drugs and enhance oral permeability [46]. Among these, SGC is used widely as it exhibits less toxicity, enhances protease enzyme-inhibiting potential in the gastrointestinal system, and improves the permeation effect [47,48].

Solid lipid nanoparticles (SLNs) are colloidal carriers developed as an alternative system to other existing traditional carriers (emulsions, liposomes, and polymeric nanoparticles). They are a new generation of submicron-sized lipid emulsions where the liquid lipid (oil) has been substituted by a solid lipid. The drug-loading capacity of conventional SLNs is limited by the solubility of drug in the lipid melt, the miscibility of the drug melts and lipid melt, the chemical and physical structure of the lipid matrix, and the polymorphic state of the lipid matrix [49]. If the lipid matrix consists of especially similar molecules (i.e., tristearin or tripalmitin), a perfect crystal with few imperfections is formed. Since incorporated drugs are located between fatty acid chains, between the lipid layers, and also in crystal imperfections, a highly ordered crystal lattice cannot accommodate large amounts of drug. Therefore, the use of more complex lipids is more sensible for higher drug loading. Thus, potential disadvantages such as poor drug loading capacity, drug expulsion after polymeric transition during storage, and relatively high water content of the dispersions (70–99.9%) have been observed. Release can be controlled depending on where the drugs are incorporated within the particle (solid solution model and core–shell model with a drug-enriched shell or a drug-enriched core; Figure 1). When ionizable lipids are used, they can complex genes; moreover, as they are usually neutral at physiological pH and charged in acidic endosomes, they encourage endosomal escape for intracellular delivery of genes [50,51]. There is no need for organic solvents in the production of solid lipid nanoparticles, which excludes the toxicity risk resulting from solvent residues. Furthermore, the large-scale manufacturing and great reproducibility of lipid nanoparticles are vital characteristics for clinical applications [52]. Sharing advantages with SLNs, nanostructured lipid carriers (NLCs) which are made with unstructured lipid blends of liquid and solid lipids able to form an imperfect crystal internally, as well as possess improved drug loading and enhanced stability over storage, as the liquid phase prevents the release of drugs during storage [53].

Cochleates are small-sized and stable lipid-based carriers comprising mainly of a negatively charged lipid (e.g., phosphatidylinositol, phosphatidylserine, phosphatidylcholine, and diolylphosphatidylserine) and a divalent cation such as calcium with a cigar-shaped spiral multilayered structure [54,55,56]. Hydrophobic, amphiphilic, and negatively or positively charged molecules have been delivered by cochleates and are ideal candidates for oral and systemic delivery of hydrophobic and hydrophilic drugs prone to oxidation [56], enabling an enhancement in permeability and a reduction in the dose of drugs. Divalent cations are generally used for rolling of lipid sheets and interacting with the lipids which are present on the outer membrane of the cells [57]. Cochleates show many applications such as oral delivery of amphotericin B (AmB) for leishmaniasis, cochleates for antibiotic resistance, antigen transportation for treatment of meningitis B, encapsulation of volatile oil for leishmaniasis, and topical application for antifungal applications [56].

Lipid-based nanomedicines can enable passive (based on their size and enhanced permeation and retention effect observed in tumors as in the case of Doxil^®^) or active targeting by modifying their surface with ligands able to bind specific receptors (e.g., EGFR, Transferrin, HER-2, and asialoglycoprotein receptors [58]), which allows beneficial biodistribution and tumor/tissue accumulation tailored to the indication [59,60]. Liposomes, however, remain over the last three decades the nanomedicines that resulted in the majority of approved therapeutics [7,61,62,63] for multiple applications such as oncology, pain, and infection, while liposomes are well represented in current clinical trials for chemotherapy, gene therapy, and vaccination (Table S1 [64,65,66,67,68,69,70,71,72,73,74,75,76,77,78,79,80,81]).

Recently, therapeutics based on nucleic acids, including small activating, interfering, and messenger RNAs (saRNA, siRNA, and mRNA, respectively) have received interest for a broad range of diseases and infections [82,83]. However, there are some inherent drawbacks with using nucleic acids, such as low immunogenicity of DNA and the possibility of its integration with the human genome [84,85], rapid degradation of RNAs in physiological environments, and their excretion within a short time (<10 min) by glomerular filtration [86]. Lipid nanomedicines are emerging as formulations able to reduce serum endonuclease degradation, as well as to target the genetic medicines to the cells/tissues required [87]. Various lipids possess robust self-adjuvant activity, particularly cationic lipids (e.g., dimethyldioctadecylammonium bromide), which enables antigen deposition at the injection place, while improving intracellular delivery and complexation of antigens [88]. However, the level of immunogenicity is highly dependent on the formulation type (i.e., lipid nanoparticles showed high antigen complexation and cell uptake, while emulsion-based systems indicated elevated antibody responses) [88]. In another study, mRNA lipid nanoparticles with adjuvants (tri-palmitoyl-*S*-glyceryl-cysteine (Pam(3)Cys) bound to the pentapeptide) were able to elicit synergistic effects in cancer immunotherapy [89]. Various TLRs (Toll-like receptors) were triggered by this formulation to enhance the CD8^+^ T-cell population required to limit tumor growth [89].

Since the outbreak of the COVID-19 pandemic, vaccines based on mRNAs have revolutionized vaccination, enabling shorter research and development cycles, simple manufacturing procedures, and the capability of intense immune response induction. Currently, most COVID-19 vaccine candidates based on mRNAs employ lipid-based nanoparticles (LNPs) as a delivery vehicle formed from four elements, including helper phospholipids (e.g., oleoylphosphatidylethanolamine or dioleoylphosphatidylcholine), cholesterol, PEGylated lipids, and ionizable lipids. More than 300 vaccine candidates for the COVID-19 pandemic were reported to be under development by the WHO, of which 47 were vaccines based on mRNAs, among which 23 have entered clinical trials [102,103]. Pfizer–BioNTech was the first officially approved COVID-19 vaccine on 23 August 2021 by the FDA for commercialization [104], being also the first-ever approved vaccine for emergency use in children 5 through 11 years old [105]. Additionally, liposomes have also been used for vaccination as adjuvants as in the case of Shingrix, Mosquirix, Epaxal, and Inflexal V, which are four approved and successfully commercialized liposomal vaccines [62,63,88]. These vaccines offer several advantages compared to vaccines based on conventional proteins, such as high safety, ease of synthesis, efficient manipulation of antigens, low cost, and having the capability for scaling up [85,106], while they offer advantages in terms of their pharmacokinetics, ability to protect the genetic material, and capability of targeted and intracellular delivery (macrophages and dendritic cells), as well as tissue distribution [81,84,107,108,109]. Similar results were also shown for anticancer vaccines [86,107,110].

### 2.1. Current Methods for Lipid-Based Nanomedicine Manufacture

Lipid-based nanomaterial fabrication can be categorized as organic solvent injection, hydration, reverse-phase evaporation, and detergent removal methods [111,112,113,114,115]. The classic manufacturing techniques for nanomedicines and in particular for liposomes are labor-intensive and suffer from a number of difficulties in their application at an industrial level, e.g., poor reproducibility and insufficient cost-effectiveness (Table 2) [116,117,118]. Batch synthetic methods for liposomes are generally based on specific parameters to guarantee the self-assembly [117]. Lipid-based nanoparticles using injection of organic solvents can be fabricated in a single step. In this method, lipid concentrations, mixing rate, injected volume, ratio of aqueous solution, and solvent/lipid ratios are the main variables to control the size of produced nanoparticles [119]. The hydration technique is the most conventional procedure for manufacturing of large multilamellar vesicles (100–1000 nm). Briefly, a lipid film is fabricated via an organic solvent evaporation from the lipid–solvent solution in a flask or tube; subsequently, an aqueous solution (e.g., phosphate-buffered saline) is added to form multilamellar vesicles. The vesicles fabricated by experiencing extra size tuning processes (e.g., sonication and extrusion) are turned to unilamellar small vesicles (<100 nm) [120,121]. The size of the vesicles is optimized in terms of applied power (sonication) and pore sizes of the employed membrane (extrusion). After fabrication of unilamellar small vesicles, a freeze/thaw method is used for drug loading in lipid-based nanoparticles [122]. The detergent removal technique works on the basis of vesicle formation (lipid molecules and detergents) and detergent elimination by dialysis [123]. As aforementioned, these fabrication approaches commonly contain three main steps: dissolution of lipids in organic solvents, lipidic phase dispersion in aqueous media/solution, and purification of the resulting samples (e.g., liposomes and nanoparticles) using some complex methods (i.e., centrifugation and/or gel permeation chromatography). These methods mainly yield large uni/multilamellar vesicles; hence, further steps (e.g., ultrasonication, high-pressure homogenization, or extrusion) are required to manufacture small unilamellar vesicles with low polydispersity index. The drawbacks of all these conventional manufacturing techniques are the use of volatile organic solvents in large quantities, the complexity scaling up, the heterogeneity of the prepared products, the high cost of excipients, and the need for multiple time-consuming steps.

### 2.2. Challenges with Lipid-Based Nanomedicine Manufacture and Clinical Translation

One of the main challenges in the field of nanotechnology has been the lack of continuous and easily scalable method for the controlled manufacture of nanomedicines with critical quality attributes (CQAs) such as size, size distribution, drug loading, surface charge, surface density of ligands or decorated polyethylene glycol chains, and stability, able to ensure batch-to-batch reproducibility. Absence of protocols and access to facilities for product characterization, as well as challenges in scale-up and good manufacturing practice, along with lack of well-trained industrial staff, contribute to delays in uptake of these technologies by the pharmaceutical industry [17,124]. Although academics possess the necessary skills and knowledge to develop these systems, the lack of business management education at academic level contributes toward challenges in their industrial and clinical uptake. The absence of proper controls, inadequately outlined critical quality characteristics, and the lack of animal models with adequate clinical relevance to humans that actually mimic the action mechanisms of nanomedicines in the body have limited extensive clinical translations. The restrictions enforced by too complicated models or too simplistic procedures that impede reliable data interpretation emphasize that there is a need for stratification and standardization of methodologies [125]. Nanomedicines are not formally controlled and organized differently from conventional small therapeutics. To be effectively translated into the healthcare market, the EMA and FDA both ask that nanomedicines satisfy the same efficacy, safety, and pharmaceutical characteristic standards used for all therapeutic products [126]. However, because of the hybrid and unique nature of nanomedicines, the quality evaluation of these products shows considerable analytical challenges in comparison with small biological (e.g., antibodies) or molecular drugs. In addition to the identity, potency, strength, impurities, stability measurements, bioburden, and bacterial endotoxins of various chemical ingredients, further physicochemical characteristics and sterility must be evaluated for the final nanomedicine. These evaluated characteristics include size distribution, particle size, polydispersity, drug loading, surface charge, drug dissolution behavior, complex core/shell physical and chemical structure, size, and chemical stability while in storage or contact with biological environments [127]. Classical characterization approaches are usually not able to be used to assess nanomaterials, and more advanced methodological techniques are required to realize how nanomedicine characteristics could affect their efficacy and safety profiles (e.g., assessed by their biodistribution, pharmacokinetics, immunological effects, degradation profile, and metabolism) to identify the essential quality features of each system [128,129]. Thus, the lag with respect to regulatory guidance hinders the progression of nanomedicines in clinical development.

## 3. Microfluidic Manufacture and the Problem of Mixing

The current manufacturing methods for the majority of licensed nanomedicines remain discontinuous and face a number of problems such as high batch-to-batch variability affecting the CQAs of the product, laborious multistep processes, need for an expert workforce, and not being easily amenable to industrial scale-up involving typically a complex process control [1]. Inability to control the CQAs for nanomedicine is linked to poor control of bioequivalence that invariably results in poor therapeutic efficacy. The FDA also supports transforming batch to continuous manufacturing processes to improve product quality and reproducibility, which would also be less labor- and time-intensive [130].

Several techniques have emerged in recent years for nanomedicine manufacture; however, a paradigm shift occurred when microfluidic strategies were employed. Microfluidics is the technology of fluid manipulation in channels with dimensions of tens of micrometers [131,132], and small volumes of liquid reagents are rapidly mixed in a microchannel in a highly controlled manner to form nanoparticles with tunable and reproducible structure that can be tailored for drug delivery, resulting in a continuous and industrial amenable manufacturing process. Largely irrespective of the nature of the process, continuous flow conditions offer clear advantages over traditional batch processes, as quantity of the product scales directly with time but does not require different reactors (easy scalability), while fixed geometries allow for a precise control of mixing conditions (reproducibility) and enable lower size dispersity, as well as, in some cases, better drug loading; moreover, fine-tuning of particle properties such as a size is possible via control of the process parameters such as flow [133,134,135,136].

### 3.1. Microfluidic Devices and Principles

#### 3.1.1. Principles of Mass Transfer and Fluid Mixing

Theoretically, in microfluidic environments, the fluid flow is controlled by the same rules governing the flow of a fluid at the macroscale. Microfluidic devices are not simply a miniaturized type of their macroscale versions, due to several physical features (e.g., high ratio of surface/volume and mass transfer based on diffusion) that do not linearly scale from macrodomains to microdomains. Microfluidic systems are described by the ubiquity of laminar flow, because of the controlling role applied by viscous forces [137,138,139,140]. It is important to remember that microfluidic mixing due to the small lateral dimension of the channels causes the flow to be laminar as the Reynolds number (*Re*, Equation (1)) is inevitably an order of magnitude lower than the minimum necessary to achieve turbulence (*Re* >> 10^3^).

(1) $$Re=\frac{Vdp}{\eta },$$

where *V* is the flow rate, *d* is the diameter of the channel, *p* is the density of the fluid, and *η* is the viscosity. Increasing the *Re* cannot be only increased by a large increase in flow rate, as this would significantly increase the pressure and flow rate while decreasing channel diameter. Thus, where the flow is laminar in the fluidic domain, mass transfer is governed by passive molecular advection and diffusion [1,139,140]. Mixing at the macroscale is commonly obtained via the formation of turbulent flow, enabling it to separate fluid in small parts, thus resulting in a decrease and an increase in the mixing path and contact surface, respectively. Architecture of a micromixer is usually designed in such a manner to reduce the path of mixing and enhance the contact surface region. As mixing is based on diffusion, the mixing time (t_mix_) is proportional to the square of the width of the fluid stream (d) and inversely proportional to the diffusion coefficient (*D*). The latter is inversely related to size (hydrodynamic radius of the particles), which means that it is slow for polymers, and this can lead to more thermodynamically stable products such as microparticles with lower interfacial energy than nanoparticles due to a slower nanoprecipitation process as shown by the Einstein–Stokes equation (Equation (2)).

(2) $$D=\frac{kT}{6\pi \eta R},$$

where *D* is the diffusion coefficient for a particle in a free volume, *k* is the Boltzmann constant, *T* is the absolute temperature, *η* is the viscosity of the solution, and *R* is the hydrodynamic radius of the particles. Considering the diffusion coefficient for poly(ethylene glycol) (PEG) 1 kDa polymer (*D* ≈ 3 × 10^−10^ m^2^·s^−1^) in water, the solution would cover 100 µm in 30 s which would need a flow rate of 1 mL·min^−1^ in a channel that would be at least 15 cm long.

**Figure 2:**
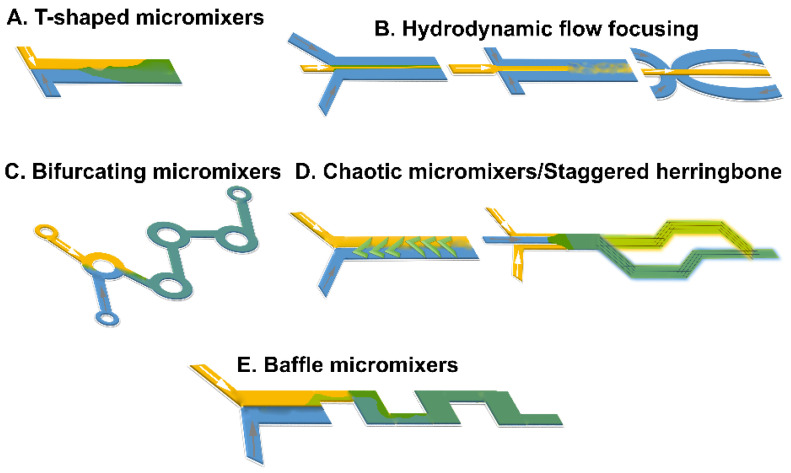
**Summary of schematic designs of microfluidic mixers for lipid nanoparticle development**: (**A**) T-shaped mixer, (**B**) hydrodynamic flow focusing, (**C**) bifurcating mixers, (**D**) chaotic, staggered micromixers, and (**E**) baffle mixers.

Often, materials and solvents are chosen to maximize the reciprocal diffusion coefficient and to minimize viscosity. Typically, the geometry and nature of the flow are designed to act on the area of convergence of the different fluids or the area immediately after (mixing region) (Figure 2). Hydrodynamic flow focusing (HFF) devices focus on the confluence point and control the width of a central flow that carries the material of interest and is enveloped by lateral flows. The second type aims to transition from a laminar to chaotic flow. Although this can be achieved by curvilinear channels, passive micromixers typically have paths with complex and tortuous shapes. Heterogeneity in the flow itself, e.g., by introducing high-molecular-weight polymers that alter microviscosity of the liquid, can also contribute toward achieving chaotic mixing [127,128]. Static mixer efficiency is usually compared via the Peclet number (length of channel) as it is indicative of the ratio between mass transport through convective (chaotic flow) and diffusive flux (laminar flow) and is calculated using Equation (3).

(3) $$Pe=\frac{vl}{D},$$

where *ν* is the velocity of the fluid, *l* is the characteristic length of the fluid, and *D* is the diffusion coefficient. Micromixers are regularly categorized as being active or passive, subject to the used mechanism for the formation of mixing processes at the microscale. Active devices introduce chaotic features by exploiting exterior energy powers and energy of the fluid pumping, to make time-restrained perturbations of the flow field and expedite the mixing procedure (Figure 2) [129]. According to the type of external force used, micromixers can be subdivided as driven by ultrasound energy (acoustic/cavitation) [141], pressure field [142], or magneto-hydrodynamics [143], or induced by temperature [144]. These micromixers have typically higher mixing yield in comparison with passive micromixers [145]. However, the application of these devices in practical situations is limited due to the necessity of integrating the system with secondary equipment (i.e., actuators for an exterior energy source) and the expensive and laborious manufacturing processes. Additionally, the application of external energy powers (e.g., ultrasonic waves) may lead to the formation of high-temperature gradients, which can possibly destroy involved or loaded bioactive molecules. Therefore, these mixers are not a common option when using microfluidics to chemical, pharmaceutical, and biological applications [145]. Passive mixers are the leading microfluidic devices due to the ease of their manufacturing methods and associated cost-effectiveness in comparison with active micromixers. The mixing time reduction is obtained through various approaches, including focusing fluid flows using hydrodynamic principles [146], fluid stream splitting benefiting from parallel or serial lamination [147], increasing chaotic advection employing designed groves and ribs on walls of the channel [148,149], and introducing bubbles of liquid (droplet) or gas (slug) into the stream (Figure 2) [150,151]; these were previously summarized thoroughly [152]. Although the geometry of the channel is critical in the mixing and, thus, nanoprecipitation, the engineering of microfluidic devices remains complex and available to limited manufacturers for microfluidic devices. Recent attempts have utilized 3D printing to enable the production of easily tailored geometries toward the production of microfluidic devices for the manufacture of nanomedicines [1,153].

#### 3.1.2. Microreactor Design and Mixing

A quick mass/heat transfer can significantly enhance the controllability of the mixing process that subsequently defines the physicochemical characteristics of the manufactured nanomaterials. Considering the mixing method and device features, microreactors for production of nanomaterials can be categorized into two types: segmented and continuous microreactors (Figure 2).

##### Microreactors with Continuous Flow

Microreactors with continuous flow in comparison with segmented flow are usually recognized by higher efficiency and the feasibility to continuously alter the composition of reactants through the reaction channel [154,155,156]. Accordingly, it is practicable to obtain multistep procedures by linking various reactors in series [157]. As the stream pattern is simple, scaling up can be obtained by easily enhancing the rate of used flow [158]. These microreactors can also be grouped into three main subtypes based on their microchannel network architecture (i.e., micromixer, coaxial flow, and capillary tube).

Capillary tube devices have the simplest configuration and are made of polymer [159], steel [160], or silica [161] capillary tubes with the lumen diameter of channels in the micron range, where an enhanced nanoparticle yield can be obtained through quick and precise temperature control. Their easy operation and production, along with the feasibility to employ robust materials, make them capable of tolerating the necessities of high-temperature applications, increasing the interest in capillary devices for manufacturing of nanocrystals of semiconductor and metallic nanomaterials. However, there are risks of chemical adhesion to the surface of channels, lumen blockage, and comparatively high polydispersity in products in the application of these devices [161,162]. To tackle these challenges, microreactors with coaxial stream have been designed [157,163]. Flögel et al. applied a silicon continuous flow microreactor for peptide synthesis and showed that the employed microreactor not only enables scanning the reaction conditions quickly, but also empowers the procurement of synthetically appropriate amounts of peptides [164]. It was demonstrated that coupling of peptide with 9-fluorenylmethoxycarbonyl (Fmoc)- and *tert*-butyloxycarbonyl (Boc)-protected amino acids was achieved at 120 °C in 1–5 min, and a further improvement in synthesis efficiency of β-peptides was also achieved via the application of a fluorous benzyl tag [164]. The ability to undertake couplings within the chips remains a desirable feature if functionalized particles are intended as similar chemistry is employed. In microreactors with coaxial flow, the direct contact of the reaction mixture with the channel walls are prevented by an ensheathing stream to minimize adhesion to the walls and clogging of the channels, while eliciting nanomaterials with reduced polydispersity, as the stream comprising the precipitating species is at the center of the channel center where the fluid velocity is more homogeneous compared to the flow near the channel walls. This results in a more homogeneous residence time distribution for the growing nanomaterials inside the microreactor, and various growing nanomaterials have a similar growth time within the process [157,163]. Lipid emulsions injected in flow-focusing microfluidic chips were also shown to be able to entrap microspheres, proteins, and cells [165]. A dispersed phase of aqueous solutions consisting of cells, microspheres, or proteins was sheared by the continuous phase of dissolved phospholipids in oleic acids to produce stable lipid emulsions. The prepared emulsions were injected into a mixture of ethanol and water that was an appropriate solvent for oleic acid. Forcing phospholipids in the acid resulted in rearrangement at the emulsion surface toward lipid particles due to rapid dissolution of the oleic acid into the ethanol. The encapsulated cells remained viable, and the efficiency of encapsulation depended on the flow rate of the continuous phase and on the ethanol concentration in the mixture to eliminate excess oleic acids [165]. In addition to lipidic particles, this was also applied to polymersomes [166]. Double emulsions with a core–shell structure (aqueous cores) were manufactured using a flow focusing chip and subsequently dispersed in a continuous phase of water containing glycerol (80% *v*/*v*). The emulsion shell was a layer of the cosolvent mixture of toluene and tetrahydrofuran containing the di-block copolymers of poly(*n*-butyl acrylate)/poly(acrylic acid). As tetrahydrofuran was exceedingly miscible with water, in the outer layer of the double emulsions, the cosolvent diffused into the continuous phase, resulting in the self-assembly of di-block copolymers on the double emulsion concentric interfaces [166]. Polymersomes with a stable membrane and a uniform size were formed when the evaporation step was completed. Evaluating membrane permeability revealed that the polymersomes with 1.5 μm thick walls were comparable in permeability to those with a thickness of ~10–20 nm. This finding showed the possibility of thickness inhomogeneities in the manufactured vesicles (membranes) [166]. A similar microfluidic approach was used to fabricate biocompatible monodisperse polymersomes with a membrane of poly(ethylene-glycol)-*b*-polylactic acid [167] that showed that the release of the encapsulated hydrophilic fluorescent solute could be affected by osmotic pressure differences. They studied the formation process of di-block copolymers with various molecular weight ratios of the hydrophobic and the hydrophilic blocks, such as PEG(5000)-*b*-PLA(1000), PEG(1000)-*b*-PLA(5000), and PEG(5000)-*b*-PLA(5000), which revealed that the properties of the polymersomes could be altered by incorporating various homopolymers and altering the hydrophobic and hydrophilic block ratio [167].

MHF microfluidic techniques have been shown to produce uniformly dispersed liposomes and allow for the direct control of liposome size via fine adjustments to either the flow rate ratio (FRR) or the total flow rate (TFR) [168]. A micromixer with basic channel configuration (i.e., Y-shaped) was applied to fabricate hydrocortisone (a drug with poor water solubility) nanosuspensions [169], boehmite and barium sulfate nanocrystals [158], and cadmium sulfide [170]. However, these have not been used for lipid-based nanoparticles apart from studies that utilized Y-shaped mixers incorporating staggered herringbone elements to induce chaotic advection [171,172], where the resulting liposome size correlated with the FRR in the microfluidics process (~50 nm), and high-throughput manufacturing of liposomes of similar CQAs was possible by increasing fourfold the volumetric flow rate [171]. Microfluidic hydrodynamic focusing (MHF) T-shaped chips and coaxial geometries were used for the one-pot synthesis of injectable size liposomes [173]. Narrowly distributed unilamellar nanoliposomes (~85 nm, polydispersity index of 0.13) with a composition similar to that of Doxil^®^/Caelyx^®^ could be synthesized at production rates 15–20 times larger compared to T-shaped MHF chips, and the size depended on the Reynolds number (5–50) in the coaxial configuration due to viscosity-induced mixing dynamics at the water–ethanol interface [173].

##### Microreactors with Segmented Flow

Microreactors with segmented flows can be divided into multiphase (liquid–liquid) stream or slug (gas–liquid) stream microfluidic devices [174,175,176]. A significant variable, which impacts on the monodispersity of the synthesized nanomaterials is the residence time distribution (the average spent time in the reactor). In microfluidic reactors with a laminar flow, the parabolic stream profile (i.e., slower fluid movement near the walls of channel compared to that of the fluid in the center of the channel) and the accompanied axial dispersion result in a difference in residence time that subsequently causes a broader distribution in the size of the prepared nanomaterials [177]. This issue in reactors with a laminar flow can be addressed by employing microreactors with a segmented stream, which result in a proper control on the size and size distribution of nanoparticles. This is due to slugs (gas–liquid) or droplets (liquid–liquid) that can act as a microscale reactor and flow through the channel during the process time (this is only determined by the rate of the flow). In these microreactors, mixing is obtained by leveraging the microflow produced inside the droplet or slug while it is streaming through the winding and straight channels [177]. In other words, extremely short residence times can be established using this approach, and the point of particle formation can, therefore, be better defined. The principle of flow focusing in microchannels has been used to successfully precipitate organic nanoparticles [178,179,180]. An additional compartmentation in droplets or plugs can suppress the free convection, and particle growth is controlled by diffusion and convection within the nanoliter compartments [181]. The segmentation of the continuous flow by injection of gas creates Taylor flows with plugs in which a recirculating convection occurs [182]. Accordingly, the mixing is intensified. Different studies have investigated the enhanced mixing processes in these two-phase flows [183,184]. Nevertheless, Taylor flows in microfluidic systems with separated flow focusing and gas displacement can become unstable, and nonperiodic tear-off in the gas bubbles impedes the control of plug volumes and mixing. Furthermore, in continuous flow focusing systems, nanoparticles precipitate immediately and can stick to the channel walls, leading to fouling jeopardizing stable operation and small particle sizes. Slug-flow reactors have the advantage of easy gas separation from the final reaction medium. Thus, there is no need for further post-purification processes. However, the process should be conducted very carefully in limited volumes to obtain a steady pattern of the multiphase gas flow [158]. The first microfluidic system based on droplets for producing unilamellar liposomes was studied by Tan et al. [165]. This study showed that proteins, cells, and beads could be efficiently encapsulated in liposomes with 27 to 55 µm in diameters and could facilitate ion exchange between the external environment and inner compartment [165]. This approach was also used to fabricate size controlled segmented wormlike micelles by polystyrene-*block*-poly(4-vinyl pyridine) self-assembly [185]. Comparing the assembly of these micelles with off-chip assembled block copolymers at the same solution characteristics revealed that the prepared segmented wormlike micelles were thermodynamically metastable structures and kinetically controlled assemblies, which were created by the aggregation of preformed spherical micelles in an ordered manner due to the quick mixing procedure in microfluidic channels. Furthermore, by altering the total flow velocity or the flow velocity ratio of the block copolymer and water solution, both the sizes and the percentages of segmented wormlike micelles among the whole assemblies were effectively controlled [185]. Additionally, microfluidic approaches have been employed to produce lipid vesicles (layer-by-layer asymmetric). Matosevic et al. developed an assembly-line procedure with the capability to perform a completely reproducible and parameterized phospholipid vesicle manufacture [186]. The feasibility of a flow focusing device for the fabrication of droplets and the subsequent (phospholipid) stabilization was later demonstrated as multilamellar asymmetric vesicles were formed by droplets trapping within pockets and by gradually exchanging the continuous phase with a secondary phase, including various types of phospholipids that can be deposited on the formerly created bilayer [187]. A symmetric design of a segmented flow device with the capability to combine flow focusing and segmentation was also described in which the backflow of liquid into the gas channel is suppressed, avoiding destabilization of the injected gas bubble that causes premature precipitation [188]. Consequently, the symmetric design not only widened the range of stable Taylor flows, but also allowed operation for longer periods without severe fouling [188]. Tuning the time allowance for complete mixing down to 9 ms was achieved, and lipid nanodroplets with tunable sizes down to 74 nm were fabricated [188]. However, fouling was observed when an ethanolic Softisan^©^ 100 was used as the mixture of triglycerides crystallized within the channel. Deposition of lipid material in a segmented flow micromixer could be reduced through a modification of the design [189]. The investigated segmented flow micromixer was fabricated from 700 µm thick glass wafers and had a symmetrical design with three inlets for the aqueous and ethanolic liquid phase, as well as for the gas phase, in diameters of 193 µm, 87 µm, and 146 µm, respectively. Castor oil and glycerol monooleate (monoolein) were used in this study due to their good solubility in ethanol (>100 mg·mL^−1^) [189], resulting in nanoemulsions with a droplet size between 120 and 200 nm and polydispersity indices of 0.14, when the surfactant was included via the aqueous phase, or smaller sizes when the surfactant was included via the ethanolic phase.

##### Micromixer Channel Dimensions and Residence Time Effects

Microreactors, in addition to the ability to effectively control the characteristics of prepared nanomaterials dimensionally, can be used to control and study the fundamental reaction procedures in the formation of nanomaterials [155,190]. Several methods, including small-angle X-ray scattering, spectroscopy, and spatially resolved photoluminescence imaging, are employed to study the kinetics of nanoprecipitation [191,192,193,194,195]. Continuous flow in microscale channels allows precise temporal and spatial control of reactions via the addition of reagents at predetermined time intervals within the reaction process. These characteristics allow microfluidic devices to enable pre-/post-treatments, as well as multistep synthetic processes within the reactor. A stream of lipid mixture was hydrodynamically focused at a microchannel cross-junction between two aqueous buffer streams. The formation of liposomes was energetically favorable at points in the system where the concentration of the mixture of isopropyl alcohol and buffer solution reached a critical condition where lipid solubility was low [196], resulting in liposomes (100–300 nm). Furthermore, the effect of mixing performance on the size of lipid nanoparticles using microfluidic methods was studied by Maeki et al. [196] using chaotic micromixers with various depths (i.e., 11 and 31 μm). LNPs with the smallest size and a narrow particle distribution were formed in channels of 31 µm. The size of LNPs could be tuned within 10 nm by ensuring optimum residence time and critical ethanol concentration. The critical ethanol concentration range was estimated to be between 60% and 80% according to laser scanning confocal microscopy. The residence times at the critical concentration necessary to control the LNP size were 10, 15–25, and 50 ms timescales for 30, 40, and 50 nm-sized LNPs, respectively [196].

#### 3.1.3. Heat Transfer and Temperature Control

Temperature is one of crucial factors that needs to be considered during nanomedicine fabrication as it can affect supersaturation, solubility, and kinetics. Microfluidic devices include channels with typical diameters around 10 to 1000 μm with an enhanced ratio of surface to volume (~ 10,000 to 50,000 m^2^·m^−3^) in comparison with macroscale channels (~100 to 2000 m^2^·m^−3^) [138,197]. Microfluidic devices usually show high efficiencies of thermal transfer, and this enables their use for high temperatures and/or exothermic reactions in a controllable and effective (isothermal) way [140,185,186], thus offering effective temperature control in chemical synthesis or functionalization reactions using continuous flow reactors [155,198]. The high surface-to-volume ratio speeds up heat exchange; for example, ~0.4 s is required for a channel of 200 μm diameter to increase the temperature of a liquid from 20 °C to 300 °C [199]. Temperature has multiple effects such as (i) by changing the free energy kT and the diffusion coefficients, (ii) by changing the viscosity, and (iii) by changing the membrane elasticity at or below the transition temperature and by changing the line tension. By modifying three parameters (i.e., volumetric flow rate ratio of the buffer to alcohol, phospholipid acyl chain length, and temperature) using a microfluidic hydrodynamic focusing approach, studies showed that liposomes formed at temperatures below the transition temperature of phospholipids had the largest size compared to those formed at a temperature closer to transition temperature of the lipids [200]. The larger size of liposomes at lower temperatures was due to the membranes having a much higher elastic modulus below the transition temperature. For the liposomes formed at temperatures lower than the transition temperature (e.g., ≤40 °C for 1,2-distearoyl-*sn*-glycero-3-phosphocholine or ≤10 °C for 1,2-dipalmitoyl-*sn*-glycero-3-phosphocholine), the stream of alcohol in the focusing region was not stable and slowly grew over time [200]. At these temperatures at the alcohol–buffer interfaces, large visible aggregates formed in the focusing region. At the bottom and top of the channel, these aggregates were likely the reason behind the unsteady focusing resulting in more polydisperse and larger liposomes at lower flow rate ratios. However, even at low temperatures, smaller liposomes were formed at higher flow rate ratios, although they were still bigger than the liposomes formed at higher temperatures at the same flow rates. Consequently, all tested liposome compositions through this work could produce liposomes using high flow rate ratios at or above room temperature; however, at room temperature, liposomes prepared with 1,2-distearoyl-*sn*-glycero-3-phosphocholine were less reproducible because of flow fluctuations and aggregations in the focusing region [200]. It was shown that the size of the liposomes was decreased in a microfluidic process with decrease in needle diameter (or increase in hydrodynamic pressure), decrease in lipid concentration in the alcohol solution, decrease in phase transition temperature (T_m_) of the lipid bilayer, and absence of cholesterol (or decrease in membrane rigidity) [201].

### 3.2. Materials for Microfluidic Chip Fabrication Applicable for Nanomaterial Production

**Figure 3:**
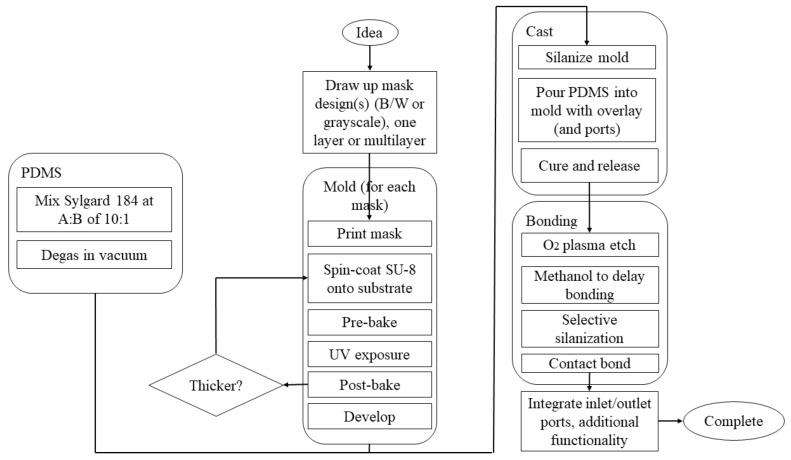
**Process of making microfluidic devices using PDMS**. Different materials are used to produce the mold, but SU-8 is usually chosen in the production of PDMS-based microfluidic devices. Once the mold has been prepared with the appropriate steps, the next step is casting, followed by hardening and release of PDMS from the mold. The PDMS is deposited on the mold; everything is placed in the oven for 24 h at 65 °C so that the PDMS cures and, once hardened, can be easily removed from the mold. Then, the bonding phase follows, where the surface of the PDMS is generally exposed to oxygen plasma for 10 min and then in contact with a layer of glass or another layer of PDMS to generate a bond. The process ends with the interfacing and integration phase where input and output zones are created with the help of needles, in the case of temporary applications, or with specific structures for longer applications. Reprinted with permission from [207]. Copyright 2022, AIP Publishing LLC.

Initial materials for the manufacture of microfluidic devices were taken from microelectronics where silicon is widely used [1,202,203,204] due to its monocrystalline structure, availability, compatibility of its physicochemical properties with a broad range of applications, and feasibility of integration with electronic circuits. Glass is used due to its desired optical characteristics, low cost, efficient dissipation of heat capability, and high resistance to chemical and mechanical stress [202,203,204,205]; crown white, quartz, borosilicate, and soda–lime glasses are the most commonly used types [204,205]. However, the amorphous structure of glasses is the main drawback of these materials due to the possibility of nonparallel wall formation during isotropic wet etching of a glass with hydrofluoric acid. The etching procedure takes place on the exposed surfaces of the glass, and, as the etching process goes further in a channel, there is a simultaneous etching on side walls, also resulting in the formation of channel geometries with low aspect ratios. To achieve a channel with a deep length, dry etching approaches (e.g., deep reactive ion etching) can be used, but this requires costly instrumentation to be processed [204,205]. The prolonged process cycles and complex instruments accompanying the microfabrication of silicones and glasses create a necessity for the development of microfluidic devices using other substances. Polymers are emerging as materials for microfluidic devices [1,138,204,206], with poly(dimethylsiloxane) (PDMS) being a preferred material due to (i) its capability to be molded (elastomeric material), patterned easily into channels, and recreate features in micro-size with high accuracy, (ii) its low water permeability, and (iii) its appropriate optical transparency. PDMS is biocompatible, has low cost and low toxicity, and remains chemically inert, showing mechanical flexibility. The soft nature of the mold has several advantages such as optimal contact between the mold and the surface without the addition of external pressures, while the porous nature allows working both with polymeric solutions and gelling because the solvent can evaporate through the mold. The soft mold, like the PDMS mold, can be used in soft lithography (Figure 3 reproduced from [207]), originating from an original hard master mold generated with other techniques [208].

However, the main drawback of using PDMS in the synthesis of organic nanoparticles is its poor resistance to organic solvents (it is swelled in the presence of organic solvents), including aromatic and aliphatic hydrocarbons, while it is dissolved in strong acids (e.g., trifluoroacetic and sulfuric acids) and amines [1,184,193,195]. PDMS chips require manual operations during manufacture and clean rooms, while manufacturing devices in series is not possible; thus, the process remains very costly with long manufacturing times [207].

For this reason, other manufacturing techniques have been developed that guarantee the production of microfluidic devices at the nanoscale with time and cost reduction, as well as the possibility to work with different materials. Alternative polymeric substances (i.e., acrylates, modified poly(dimethylsiloxane), polyether ether ketone, cyclic olefin polymer, cyclic olefin copolymer, polycarbonate, and poly(methyl methacrylate)) have recently been used in rapid prototyping approaches to create microfluidic reactors with high resistance to solvents, precise replication ability of micropatterns with high-quality surfaces, and suitability for mass production at a low cost [1,138,153,204,206]. Cyclic olefin copolymers are transparent, amorphous thermoplastics composed of linear olefins (ethene) and monomers of cyclic olefin (norbornene). In comparison with other thermoplastics, cyclic olefin copolymer has apparent advantages, such as low autofluorescence and water absorption, good optical transparency and thermal resistance, and high chemical resistance [209,210]. Due to these promising advantages, they are progressively employed as appropriate materials for the fabrication of microfluidic devices and microsystems [210]. Among the most recent manufacturing techniques, 3D printing is emerging as a low-cost and easily personalized manufacturing technique for prototypes of microfluidic devices without the need of molds and using existing materials such as cyclic olefin co-polymers and polylactic acid or photocurable resins. For instance, stereolithography 3D printers as one of the mostly used printing methods for manufacturing of microfluidic devices use photocurable polymers to fabricate a 3D structure layer by layer [153]. Objects with complex design and geometry, as well as intricate shape, can be printed using high-resolution stereolithography 3D printing [211]. Current stereolithography printing is mostly relies on photocurable resin formulations based on methacrylate or acrylate monomers and crosslinkers. These formulations quickly cure and can be affordably produced at low prices [212]. However, variable mechanical characteristics [213], shrinkage stress [214], and oxygen inhibition [215] because of their early gelation or incomplete cure are some of the potential drawbacks of this method. These challenges can be defeated using other types of resins such as epoxy resins [216], resins based on ring-opening spiro compounds [217], and composite resins [218] in the stereolithography 3D printing context. This has been extensively reviewed previously [1,138,204,206].

## 4. Microfluidic Manufacture of Lipid-Based Nanomedicines: Studies to Date

**Figure 4:**
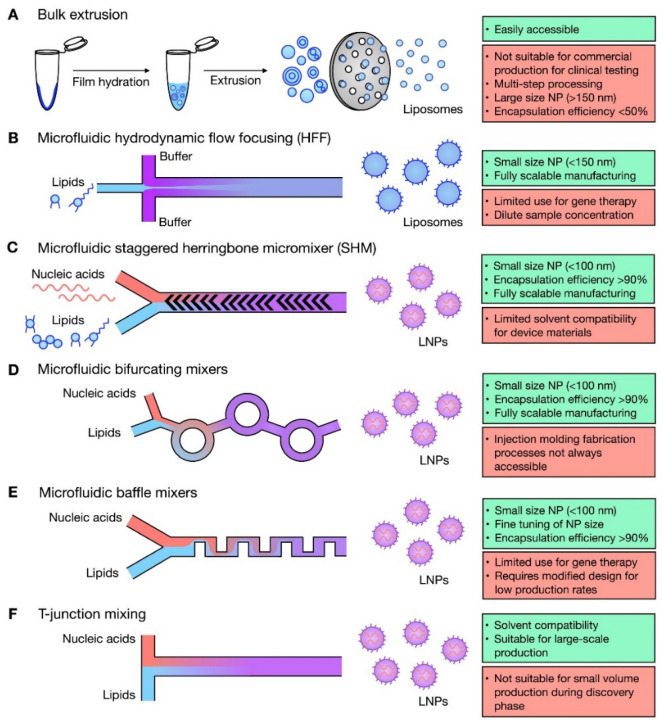
**Microfluidic techniques for liposome and lipid nanoparticle (LNP) formulation**. Summary of bulk and microfluidic techniques for production of liposomes (**A**,**B**) and lipid nanoparticles (**C**–**F**), highlighting advantages (green) and disadvantages (red) for each. Reproduced with permission from [227] and used under the Creative Commons license permission (CC BY 4.0). Copyright 2021, Elsevier Ltd. All rights reserved.

Microfluidic manufacture of lipid nanomedicines considering critical parameters such as the lipid concentration, transition temperature, total flow rate (TFR), flow rate ratio (FRR), chip geometry (chip-based or capillary-based), and purification or treatment after elution has yielded systems with controlled CQAs compared to conventional techniques [1,185,195,196,198,206,219,220,221,222,223,224,225,226]. Figure 4 summarizes the advantages and disadvantages of microfluidic and bulk techniques for manufacturing of LNPs [227].

The size of lipid nanomedicines produced microfluidically is largely dependent on the TFF and FRR, while devices able to enable chaotic mixing are able to control better the size of the particles [228,229,230]. Rapid mixing has a significant effect on the size of the particle especially if small sizes are required, and 20 nm particles have been demonstrated under high flow rates [150,231]. However, mixing performance under high flow rates is decreased in chaotic mixers due to high *Re* [150,231]. However, recent studies have shown that the size and size distribution in microfluidic manufacture using chaotic mixers does not require complete mixing to control the size of lipid-based nanomedicines of small size. [230].

**Figure 5:**
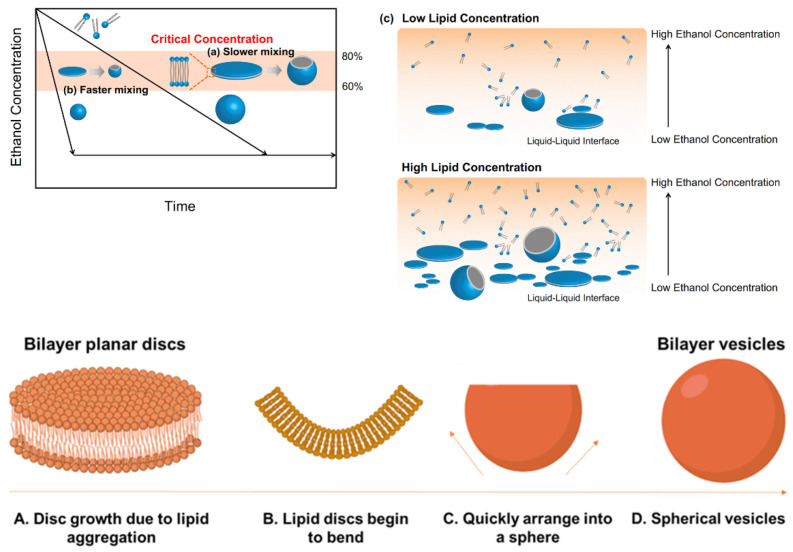
**A Schematic diagram depicting a hypothesized LNP formation mechanism**. The formation of LNPs in (**a**) slower and (**b**) faster mixing conditions. (**c**) Schematic representation of the formation of LNPs at the interface of ethanol–saline. The process starts with the aggregation of lipids in discs (**A**). The hydrophobic chains around the edges are stabilized by alcohol molecules and, as the alcohol concentration reduces, these lipid discs bend (**B**) and rapidly close (**C**) and form spherical vesicles (**D**). Thus, the polarity change during the liposome formation process is related to the initial polarity of the organic phase. The figure is adapted from **Copyright:** © 2017 Maeki et al. [196] under the terms of the Creative Commons Attribution License. All rights reserved.

The formation of lipid-based nanomedicines such as liposomes is governed by the diffusion of different molecular species such as alcohol, water, and lipids at the liquid interface between the solvent (alcohol) and nonsolvent (water in buffer) phases and is dominated by the construction of intermediate disc-like constructs, their stability at the critical aggregate concentration, and their lifetime (Figure 5) [149,196,230]. Larger particle sizes are obtained when a large amount of bilayered phospholipid fragments fuse together, typically when the diffusion of the alcohol to the aqueous phase is slow [196]. Increasing the concentration of lipid is also likely to result in larger sizes [196]. Self-assembly of the hydrophobic chain of lipids occurred due to the solution polarity enhancement as the semi-stable bilayer phospholipid fragments grew until they transformed into thermodynamically stable vesicles (i.e., lipid nanoparticles) due to an enhancement of surface energy. Subsequently, the grown bilayer phospholipid fragments were transformed to lipid nanoparticles to reduce the surface energy in the system. However, if the solvent (e.g., ethanol) is diluted quickly, the phospholipid fragments cannot grow enough to produce lipid nanoparticles (Figure 5) [196].

### 4.1. Nanomedicines Prepared with T- or Y-Junction (Shaped) Mixers

These mixers are the simplest and earliest geometric designs (Figure 2A and Figure 4) used for lipid based nanomedicines allowing for a fast mixing process [232], where an anti-solvent and solvent are combined under laminar flow, and diffusion-based mixing takes place at the interface of these two fluids [180]. In devices with Y-shaped geometry, the mixing takes place at the interface of the solvent/aqueous in the surface of the main channel, and the main factor that controls mixing is the rate of diffusion; the fluid mixing time tends to be long at quite low *Re* due to the dominating flow regime that is laminar. Consequently, by modifying the geometric design of these mixers, the capability of application of higher flow rates was created to produce perturbations that could enhance the efficiency of the mixing [233]. A broad range of mixing can be achieved using T-mixer designs with flow regimes from a laminar to a turbulent flow with *Re* in the range of 100 to 4000 [234] for nanomedicines. A segmented gas–liquid flow strategy has been proposed to improve the efficiency of mixing between two miscible liquids and to decrease mixing time, enabling mixing length shortening [150,235]. Recent studies have shown the ability to produce cannabidiol (CBD)-loaded liposomes after passive mixing using 3D fused deposition modeling (FDM)-printed polypropylene T-mixers with either a zigzag bas-relief (a 1 mm square section attached to the zigzag structure having a height of 500 µm and a total length of 60 mm) or a split-and-recombine channel shape (two square inlets at 1 mm to form a T-junction attached to main channel where circular splitting is repeated six times and kept unequal to allow a difference in the fluid velocities leading to unbalanced collisions of fluid streams with the major square sub-channel having a section of 600 µm while the minor sub-channel has a section of 400 µm) [236]. Liposomes were prepared using soya phosphatidylcholine (SPC) and cholesterol (3:1 *w*/*w*) and CBD in ethanol at two different lipid concentration (10 and 15 mg/mL) with FRR (1:3 or 1:5 ethanol/water) and a TFR of either 10 or 12 mL·min^−1^. Mixing was more efficient with an FRR of 1:3 at a TFR of 10 mL·min^−1^ and resulted in liposomes with a size <150 nm and low polydispersity (<0.15) with high loading (~73%) (Table 3) [236]. However, T-mixers alone with no further medication allow for poor control of particle size of fabricated particles and typically require a high volume of starting solutions, which limits their use in pilot studies [237].

### 4.2. Microfluidic Hydrodynamic Flow (MHF) Focusing

This configuration involves a cross-shaped flattened pattern where laminar flow predominates, and an organic solution is flowed between two streams of aqueous liquids entering from two tubes perpendicular to the organic liquid tube (Figure 2B and Figure 4) [180]. A stream of lipid in alcohol solution is forced to flow in the central channel of the device which is intersected and sheathed by two lateral or coaxial streams of aqueous phase (e.g., buffer), such that the lipid containing stream is hydrodynamically focused into a narrow sheet having a rectangular cross-section (chips with cross flow geometry) or a circular cross-section (3D annular coaxial chips) [168,180,238]. The size of the focused stream is tuned by adjusting the volumetric flow rate ratio (FRR) between the lipid- and water-phase streams and the total flow rate (TFR) [239].

The formation of lipid-based nanomedicines such as liposomes in MHF chips is governed by the diffusion of different molecular species such as alcohol, water, and lipids at the liquid interface between the solvent (alcohol) and nonsolvent (water in buffer) phases [238,240]. The reduction in alcohol concentration in which the lipids are initially solubilized by diffusion into the water and vice versa reaches a critical level below the solubility limit of the lipids, thus triggering the formation of intermediate structures (in the form of oblate micelles) that subsequently form liposomes (self-assembly, Figure 5) [200,238,241]. These devices can fabricate lipid nanoparticles with high encapsulation efficiency in a broad range of particle sizes (i.e., 30−250 nm) [242]. MHF microfluidic techniques produce uniformly dispersed lipid-based particles where the size is controlled by fine adjustments of the FRR and TFR. Decreasing the sample stream width to micrometer length can allow for controlled and reproducible mechanical and chemical conditions across the stream width that has no analogous protocol on the macroscale [243]. The microfluidic parameters that affect the particle characteristic are directly related to lipid concentration [244,245,246] and inversely related to FRR [238,239], and TFR has only a small effect on overall particle size [180,238,239]. A particle size of 30–250 nm can be obtained without the need for extrusion through the pores of polycarbonate membranes, treatment with ultrasound, homogenization, or repetitive freezing and thawing cycles [180,238].

In most studies, isopropanol (IPA) is used as the lipid solvent (Table 3); however, very few if any studies provide the residual IPA content, and few studies utilize ethanol, which is less toxic for medicinal applications [247,248]. The miscibility of the solvent with the aqueous buffer depends on its chemical structure but also on its surface tension, whereby a lower hydrocarbon chain of the solvent results in higher miscibility. Ethanol, which is almost always the solvent of choice, has a short carbon chain and is able to form hydrogen bonds with water [241]. Safe levels of the solvent in the final formulation and generally regarded as safe (GRAS) status are established according to the International Council for Harmonization guidelines (*ICH guideline Q3C (R8) on impurities: guideline for residual solvents)* [249], representing another parameter that needs to be considered (methanol; ICH class 2, limit: 3000 ppm, ethanol: ICH class 3, ICH limit: 5000 ppm, IPA: ICH Class 3, ICH limit: 3000 ppm) [241]. Studies have shown, however, that methanol–PBS (phosphate-buffered saline) results in smaller vesicles regardless of the lipid component used, while liposomes based on distearoylphosphatidylcholine (DSPC) showed an increase size in ethanol/PBS possibly due to the DSPC being more difficult to solubilize compared to other lipids [250]. Additionally, methanol and ethanol as solvents showed higher ability to load proteins compared to isopropanol [241].

The osmolarity of the buffer used and salt concentration can also affect the size of produced lipid vesicles. For cationic liposomes prepared with 1,2-dioleoyl-*sn*-3-phosphoethanolamine (DOPE) and 1,2-dioleoyl-3-trimethylammonium-propane (DOTAP), the vesicles showed an increased vesicle size from 40 to 600 nm when the Tris buffer concentration increased, while, for neutral liposomes prepared with DSPC and cholesterol, the particle size remained unchanged irrespective of the salt concentration of the buffer [251].

High drug loading can be obtained, and studies have demonstrated that, even for lipid–nucleic acid complexes, encapsulation efficiency can be improved by 20% compared to bulk mixing [242]. MHF mixers can enable the manufacture of stealth liposomes, as well as liposomes with surface modifications (e.g., folic acid as an active targeting ligand) [225]. In this work, a central flow of lipids in isopropanol that was focused using streams of PBS resulted in 55–200 nm liposomes with the size decreasing with increasing PBS-to-isopropanol flow rate ratios [225]. MHF microfluidic devices have been used for manufacturing dual-targeted liposomes functionalized with a cell-penetrating peptide and folic acid that resulted in improved targeting and extended retention in a xenograft ovarian adenocarcinoma tumor model (SK-OV-3) compared to single-functionalized or stealth liposomes alone [223]. The density of the ligands on the surface of these liposomes was independent of the FRR for the cell penetrating peptide and folic acid [223]. In particular for 3D annular coaxial chips, particles with an extremely low polydispersity (<0.05) were demonstrated along with even fourfold higher yield [252]. However, 3D annual coaxial chips require high FRR and, thus, high volumes, which can increase production costs for some therapeutics (e.g., nucleic acids) and can also lead to sample dilution that can require postprocessing to obtain desirable concentrations for preclinical or clinical studies [121]. The lipid concentration typically used for liposomes produced by MHF is relatively low with respect to liposomes present in commercial medicines (Table 3). For liposomes produced with an FRR of 10 or 30, the typical final total concentration of lipids ranges between 0.16 and 0.45 mM; however, in other techniques, this ranges between 0.1 and 2.0 mM according to FRR [168]. This limitation of MHF techniques is particularly critical when microfluidically produced liposomes are compared to liposomes for preclinical or clinical studies, where lipid concentrations range between 5 and 25 mM [253,254]. Even though MHF devices have not been employed as widely as other microfluidic platforms, they deliver remarkable benefits over traditional manufacturing methods (e.g., ethanol injection, extrusion) and can be cost-effective [168,252].

### 4.3. Microfluidic Staggered Herringbone (SHM) (Chaotic) Micromixers

SHMs are micromixer chips (Figure 2D) that induce chaotic mixing due to their asymmetric protrusions; consequently, they can process lipid nanoparticles with different sizes in a range of 20–140 nm by adjusting the FRR and TFR [149,229,255,256,257]. These chips are efficient with low-availability materials and have been used for efficient mixing, even of very low volumes of siRNAs (as low as 10 μL), which empowers screening strategies and, therefore, lipid composition identification for early preclinical studies [258]. It is important to note, however, that, as the concentration of lipids increases, the size of liposomes also increases [259]. The impact of the micromixer channel dimensions, FRR, and particle size is not as well characterized, considering that smaller particle size is achieved with higher FRR [228]. Although the micromixer channels need to have internal structure to produce sizes below 50 nm, for low FRR (≤3), micromixer channels of at least 30 µm in diameter are required as smaller channels (~11 µm) were not able to control the size. However, for FRR (≥9), both devices were able to control the size [196]. SHM cycle numbers of 10 were suggested as the limiting cycles to manufacture small-sized LNPs under all FRR conditions [230]. Increasing the contents of PEGylated lipids resulted in lipoplexes using SHM with sizes down to 20 nm and encapsulation efficiencies of siRNA above 95% [228]. Additionally, upscaling of the manufacture of these particles was shown to be feasible at a high rate by device architecture parallelization (six staggered herringbone micromixers into one chip to fabricate lipid-based nanoparticles at 72 mL/min) [228]. Lipid-based nanoparticles in smaller sizes with narrow polydispersity have been shown to be obtained by enhancing the staggered herringbone cycle number or increasing the FRR [230]; however, ten cycles have been shown to elicit particles of desired sizes with narrower polydispersity index and without the undesirable increase in FRR [230]. High pressures are associated with high FRR, and this usually adversely impacts both the chip and the pump used. When SHM strategies are combined with design-of-experiments methodologies, quick optimization of desired formulation for siRNA is possible and results in formulations that were reported to elicit to sevenfold higher expression compared to traditional preparation methods [260,261]. Studies have also demonstrated that an enhanced identification of hits is possible by combining molecular barcoding with SHM microfluidic preparation toward a library of lipid-based nanoparticles with encapsulated factor VII siRNA or identical DNA barcodes to investigate hepatic gene silencing and accumulation of particles [262,263]. SHM strategies have been used to formulate lipid-based particles loaded with poorly soluble drugs. Propofol-loaded liposomes prepared using phosphatidylcholine and cholesterol allowed aqueous dispersions of propofol of ~300 mg·mL^−1^ that were 2000-fold higher as a function of propofol’s aqueous solubility (0.15 mg·mL^−1^) [264]. These liposomes also surpassed the solubilization capacity of liposomes prepared using conventional sonication methods (120 mg·mL^−1^) [264]. However, the major drawback of using chaotic mixer devices for the production of LNPs is the possibility of their groove’s blockage by LNPs that leads to the sample flow stagnation [265].

### 4.4. Bifurcating Mixer

Although SHM micromixers have been effective in the manufacture of controlled lipid-based nanoparticles, their production rate is limited to a low TFR due to their microchannel design (Table 4), which might prove challenging in large-scale manufacture. As the recent pandemic has demonstrated, the pharmaceutical industry was only able to respond to the unmet societal needs by utilizing current technologies to rapidly optimize and enable large-scale manufacture of mRNA lipid-based vaccines as targeted and safe delivery carriers with acceptable toxicological profile. NxGen (Precision Nanosystems, San Francisco, CA, USA) has been proposed as a novel chip design based on bifurcating mixers in series (Figure 2C), also known as a toroidal mixer. The fluid flow in this device is divided and subsequently combined several times to create a rapidly mixed environment. NxGen technology is able to produce lipid-based nanomedicines at a high rate (up to 200 mL·min^−1^), while maintaining the same effectiveness of SHM micromixers and control over the particle polydispersity and encapsulation efficiency [266]. GenVoy-ILM™, a proprietary ionizable lipid mix formulation loaded with PolyA (N/P 6), was produced using different microfluidic mixers: a staggered herringbone (SHM) and a toroidal mixer (TrM) (NanoAssemblr Classic and NxGen™ respectively; Precision NanoSystems Inc., Vancouver, BC, Canada). The produced GenVoy-ILM™-based Poly (A) iLNP nanoparticles were diluted to an ethanol concentration below 1% and ultracentrifuged at 3000 rpm using 10 kDa MWCO ultrafiltration units for the removal of solvent [266]. An in vitro and in vivo investigation of gene editing using the transthyretin gene (Cas9 mRNA and sgRNA) complexed with lipid-based nanoparticles for single guide RNA delivery in murine models demonstrated that the optimized editing formulation resulted in knockdown of transthyretin protein (>97%) for a year [267]. Other studies also showed that using this technology cholesterol can be substituted with other derivatives and that β-sitosterol enabled enhanced transfection in vitro for mRNA [268].

### 4.5. Baffle Mixers

In addition to bifurcating mixer platforms, another design of microfluidic devices has been applied for the controlled fabrication of lipid-based nanoparticles and liposomes. In baffle mixers, a series of perpendicular turns are designed in pathways of a fluid to mix components of lipid-based nanoparticles more rapidly (Figure 2E) [269]. To overcome the issue of sample flow stagnation observed with SHM, a two-dimensional baffle mixer (iLiNP) was developed [269]. Lipid nanoparticles with an average size of 20 nm to 100 nm were formulated, with 10 nm intervals, by adjusting the flow rate and its ratio, as well as the device dimensions. Thus, the size could be manipulated in 10 nm intervals by adjusting the TFR and FRR and device dimensions. However, a device with a chaotic mixer structure produced LNPs sized in the narrow range of 30 to 40 nm at the same flow rate conditions (FFR 9) [269]. The authors stated that the secondary flow generation in the iLiNP device was indispensable for tuning the size of LNPs and fabricating the small-sized LNPs [269]. Factor VII gene silencing in ICR mice was found to be more than 90% at a predetermined dose of lipid nanoparticles (YSK-5 and 1,2-dimuristoyl-*rac*-glycero-3-methoxypolyethylene glycol) of siRNA (0.1 mg/kg) manufactured using a baffle mixer [269]. In general, baffle and bifurcating mixers are both single-layer chips that have demonstrated feasibility for the production of lipid nanoparticles as alternative approaches to SHM and MHF, while they are capable of being used in screening with low availability of material.

**Table 3:** Summary of microfluidic manufactured lipid-based nanomedicines.

Delivery System/Lipids	Drug/API	Chip Design	FRR (aq:org)	TFR (mL·min^−1^)	In Vitro Findings	In Vivo Findings	Optimized Formula			Ref.
							Mean Diameter (nm)	PDI	LE%	
**Lipoplex**/ DOTAP:EPC:DOPE	pDNA	FF	10:1	140 mm·s^−1^	Transfection efficacy ~10 × 10^7^ relative light units (RLU)·mg of protein^−1^	NA	~135	~2.3	NA	[270]
		Patterned walls FF			Transfection efficacy ~6 × 10^7^ RLU·mg of protein^−1^		~115	~2.0		
**Lipid nanoparticles/** mPEG-DSPC:POPC	AmB	NASHM	3:1	12	IC_50_: 0.085 μg·mL^−1^, hemolytic at ≥25 μg·mL^−1^	NA	~39	~0.115	88	[271]
		T-junction		24						
**Liposomes**/ SPC:Chol (3:1 *w*/*w*)	CBD	T-junction and zig zag or split and combine	1:5	10	NA	NA	~110	~0.13	~73	[236]
**Lipoplex/** DOTAP:DOPE:DOPC:DSPE-PEG_2000_-FolA or DOTAP:DOPE:DOPC:DSPE-PEG_2000_	siRNA	HFF	9:1	0.0167	In vitro studies on wildtype epithelial carcinoma KB cells show endosomal uptake in the perinuclear region	NA	~40	NA	~60	[242]
**Targeted Lipoplex/** DODMA:DOTMA or DCChol:EggPC:mPEG-DSPE) modified with Tf	siRNAs (LOR-1284)	HFF	5:1	0.025−2.000	siRNA complex was stable in serum for 8 h compared to 40% of free siRNA; no significant cytotoxicity in MV4-11 cells; 6.14-fold reduction in IC_50_ for siRNA complex (105.27 nM) and increased downregulation compared to free siRNA	20% of IV dose remained in plasma after 24 h (t_1/2_: 10.2 h, AUC: 5.5 h μg·mL^−1^) compared to 1% for free siRNA. (t_1/2_: 2.93 h); 3-fold increase in t_1/2_ and decreased protein expression by 86%.	~80	NA	91.5 ± 4.5	[272]
**Transferrin-conjugated Lipoplex** (Tf-LNPs-MF)/ DOTMA:DODMA:EPC:Chol:mPEG-Cho	siRNAs	SHM	3 inlets: 3:1:1	NA	Increased permeability in HepG-2 cells by Tf receptor mediated uptake	Tf-LNPs-MF-siRNA in blood was >100 ng·mL^−1^ and t_1/2_ was 25.6 h 48 h post IV vs. <10 ng·mL^−1^ and t_1/2_ of 15.1 h for free siRNA	132.6	0.129	N/A	[224]
**Lipoplex**/ DLinKC2-DMA(cationic lipid):Chol:DSPC:PEG_2000_-C-DMA	siRNAs	SHM	3:1	0.02−4.00	NA	50% silencing in hepatocytes at 10 µg·kg^−1^ in mice	28−54	<0.1	~100	[228]
**Lipoplex**/ DSPC:mPEG_2000_-DMG:Chol:range of cationic lipids	siRNAs	SHM	1:1:2 (lipids: siRNA: buffer	1.2	NA	Gene silencing potency of >90% at 1.0 mg·kg^−1^ in mice	90.5	NA	∼80	[258]
**Cubosomes**/ DOTAP:Glycerol monooleate (GMO):GMO-PEG_2000_	siRNAs	SHM	6:1	4	Gene-knockdown efficiency of 73.6% for a ρ = n_DOTAP_/n_NA_ of 3 vs. Lipofectamine (45.8%) efficiency; Up to ρ = 10, no significant damage to cell membranes.	NA	77	0.06	>90%	[273]
**Lipid nanoparticles**/ YSK05 (cationic pH-sensitive lipid):Chol:mPEG2000-DMG	siRNA	SHM	3:1	1.5	Particles with 1%, 1.25%, and 1.5% *w*/*w* and <2% mPEG (67.1, 57.3, and 53.8 nm) show high and similar gene silencing efficiencies	FVII gene silencing activity of 50% Chol-rich 1% mPEG-LNPs was higher than 3% mPEG-LNPs	32−67	NA	∼100	[274]
**Lipid nanoparticles**/ YSK05 (cationic pH-sensitive lipid):Chol:mPEG2000-DMG	siRNA	Baffle mixers	3:1	0.5	NA	YSK-LNPs showed high FVII gene-silencing activity with no dose dependency	80	0.1	>90%	[269]
**Liposomes**/ EPC:DMPC:DPPC:DSPC	Metformin (M) and glipizide (G)	NASHM	5:1	5–15	Sustained release was achieved	NA	80–90	0.11–0.22	~20 (M) ~40 (G)	[250]
**Lipid nanoparticles**/ DSPC:D-Lin-MC3-DMA:Chol:PEG-DMG	siRNA	NASHM	3:1	12	79% mRNA knockdown produced by 1 μg siRNA	15 mg·kg^−1^ (3 doses IV over 24 h) results in a 100% uptake in peripheral blood cells that remain positive until day 10; no liver toxicity or other biochemical alternation; after 10 IV doses over 35 days, luciferase signal decreased 0.75-fold, while it increased in control mice 1.6-fold; 60% knockdown efficiency of BCR-ABL by LNP-anti-BCR-ABL siRNA in sorted leukemia cells from the myelosarcoma mouse tissue	55.03	0.046	>90	[275]
**Liposomes**/ DSPC:mPEG_2000_-DSPE	Dox, ICU	SHM	10:1 and/or 16:1	5	After 48 h, ~90% of drug was released (first order); less cytotoxic to MCF-7, MDA-MB 231 and BT-474 breast cancer cells vs. free doxorubicin	NA	~100	0.2	>80	[276]
**Liposomes**/ DMPC:DPPC:DSPC	Curcumin	NASHM	5:1	17	Increased 700-fold the aqueous curcumin solubility	When co-administered with cisplatin, it enhances cisplatin’s efficacy in multiple mouse tumor models with decreased nephrotoxicity	~125	<0.2	87.7	[277]
**Nanoemulsions**/ Cold-pressed hempseed oil:lecithin:Poloxamer 188	Hempseed oil	NASHM	4:1	12	>98% Caco-2 cell viability; increased uptake by 38.2%	NA	62	0.032	> 99	[278]
**Liposomes**/ HSPC:DOPC:mPEG_2000_-DSPE	Dox	NASHM	9:1	10	Burst release (20–30%) followed by <10% release over 7 days at 37 °C or during 3 weeks storage at 4 °C	Higher tumor accumulation (5–6% dose/g) at days 1 and 4.	~50	<0.2	> 80	[279]
**Liposomes**/ EPC or DMPC or DPPC or DSPC or PS:Chol	OVA	NASHM	3:1	15	Longer chain lipids have slower release rates; burst release observed within 12 h followed by a slower release rate	NA	60–100	<0.2	20–35	[280]
**Liposomes**/ EPC:Chol	Propofol	NASHM	3:1	2	Burst release (40%,1 h) reaching 90% within 8 h	NA	~40	0.4	85	[264]
**Liposomes**/DMPC:Chol:DEPE-PEG2000:DSPE-PEG_2000_—FA or DSPE-PEG_2000_-Cys-TAT(CYGRKKRRQRRR) 55:40:3:1 molar ratio	Folic acid (FA)	HFF	16:1	28.8 µL/min	FA and TAT liposomes have 37% and 98% increased targeting in SKOV3 cell spheroids compared to TAT liposomes and FA liposomes, respectively	Improved tumor targeting and longer tumor retention (up to 72 h); 140%, 136%, and 62% higher tumor accumulation than pegylated liposomes and FA or TAT targeted liposomes, respectively	~60	<0.3	NA	[223]
**Lipoplex**/DSPC:cholesterol: DOTAB or DDAB or D-Lin-MC3-DMA:DMG-PEG_2000_,	mRNA or ssDNA, Poly A	NxGen	5:1–1:1	12−200	NA	NA	<100	<0.25	>90	[266]
**Liposomes**/HSPC:Chol:DSPE-PEG_2000_ 56:38:5 molar ratio and EPC:Chol 45:55 molar ratio	Dox	M110P Microfluidizer^®^	Pressures of 5–20 Kpsi	1–3 cycles	NA	NA	100–110	<0.2	97–98	[281]

Key: **AmB**: amphotericin B, **BT-474**: human ductal breast carcinoma cells, **Chol**: cholesterol, **DCChol**: 3β-[*N*-(*N*′,*N*′-dimethylaminoethane)carbamoyl] cholesterol, **DDAB**: DLin-MC3-DMA: (6*Z*,9*Z*,28*Z*,31*Z*)-heptatriacont-6,9,28,31-tetraene-19-yl 4-(dimethylamino)butanoate, **DLinKC2-DMA**: 2-[2,2-bis[(9Z,12*Z*)-octadeca-9,12-dienyl]-1,3-dioxolan-4-yl]-*N*,*N*-dimethylethanamine (ionizable cationic lipid), **DMPC**: 1,2-dimyristoyl-*sn*-glycero-3-phosphocholine, **DPPC**: 1,2-diplmitoylphosphatidylcholine, **DODMA**: 1,2-dioleyloxy-*N*,*N*-dimethyl-3-aminopropane, **DOL**: dolomite microfluidic system equipped with a 5-input chip (part: 3200735, Dolomite, Royston, UK), **DOPC**: DOPE: l-α-dioleoyl phosphatidylethanolamine, **DOTAP**: 1,2-dioleoyl-3-trimethylammonium propane, **DOTMA**: *N*-[1-(2,3-dioleyloxy)propyl]-*N*,*N*,*N*-trimethylammonium chloride, **DSPC**: 1,2-distearoyl-*sn*-glycero-3-phosphocholine, **DSPE-PEG_2000_**: 1,2-distearoyl-*sn*-glycero-3-phosphoethanolamine-*N*-[methoxy(polyethylene glycol)-2000] (ammonium salt); **DSPE-PEG_2000_-FolA**: 1,2-distearoyl-*sn*-glycero-3-phosphoethanolamine-*N*-[folate(polyethylene glycol)-2000] (ammonium salt), **DSPC**: 1,2-distearoyl-*sn*-glycero-3-phosphocholine, **DSPG**: 1,2-distearoyl-*sn*-glycero-3-phospho-(1′-*rac*-glycerol), **Dox**: doxorubicin, **EPC**: egg phosphatidylcholine, **FF**: flow focusing, **HFF**: hydrodynamic flow focusing, **HSPC**: hydrogenated soy l-α-phosphatidylcholine, **ICU**: isoprenylated coumarin umbelliprenin, **LE**: loading efficiency, **NxGen**: NxGen (Precision Nanosystems, CA, USA), **MCF-7**: human breast adenocarcinoma cells (hormone-positive), **MDA-MB 231**: human breast adenocarcinoma cells (triple-negative), **mPEG-CHO**: methoxy poly(ethylene glycol) aldehyde, **mPEG: DMG**: dimirystoyl-*sn*-glycero, methoxyethyleneglycol 2000 ether, **mPEG_2000_-DSPC**:1,2-distearoyl-*sn*-glycero-3 phosphoethanolamine-*N*-[methoxy-(polyethylene glycol)-2000], **NASHM**: NanoAssemblr Benchtop with SHM, **OVA**: ovalbumin, **PDI**: polydispersity, **PEG_2000_-C-DMA**: *N*-[(methoxy poly(ethylene glycol)_2000_ carbamyl]-1,2-dimyristyloxlpropyl-3-amine, **POPC**: 1-palmitoyl-2-oleoyl-*sn*-glycero-3-phosphocholine, **PS**: l-α-phosphatidylserine, **SKOV-3** cells: human ovarian cancer cells, **SHM**: staggered herringbone micromixer, **SPC**: soy phosphatidylcholine, **TAT** cell-penetrating peptide sequence: CYGRKKRRQRRR, **TF**: transferrin, **YSK05**: 1-methyl-4,4-bis[(9*Z*,12*Z*)-9,12-octadecadien-1-yloxy]-piperidine (pH-sensitive cationic lipid).

**Table 4:** Summary of microfluidic purification techniques for nanoparticles. Adapted with permission from [282] and used under the Creative Commons license permission (CC BY 3.0). Copyright 2017, Royal Society of Chemistry. All rights reserved.

Techniques	Mechanism	Separation Marker	Sizes Separated (nm)	Efficiency (%)	Throughput (mL·min^−1^)	Pros	Cons
Field flow fractionation	Asymmetrical flow FFF	Size	5–250	87–88	0.4–1.1	Very high throughput with high separation efficiency	Specific sample/solvent systems and compatible membrane
Centrifugal	Centrifugal force	Size, density	50–200	-	0.0075	High throughput, density gradient, and dilution not required	Discontinuous
Optical	Optical force	Size, refractive index, polarizability	70–1000	-	0.010–0.375	High separation efficiency	Heating and photodamage, low throughput
Affinity capture	Surface interactions	Antigenic site, hydrophobicity, charge	100	-	0.010	High capture efficiency and purity	Expensive, multiple preparation steps
Electrophore-sis	Uniform electric field	Size, charge	<50	97	0.0004	Very high separation efficiency and resolution	Flow rate change with chemistry (buffers, wall effects)
Dielectropho-resis	Nonuniform electric field	Polarizability and size	30–60	85–100	0.000009	High throughput and separation efficiency	Requires high voltage, depends on medium conductivity, very low throughput
Magnetopho-resis	Magnetic field	Size, magnetic properties	5–200	90	0.300	Very high throughput, low cost	Long time for magnetic bead antibody labeling
Acoustopho-resis	Ultrasonic sound wave	Size, density, compressibility	<200	>90	0.00043–0.00081	High separation efficiency, controlled cut off separation	Complex fabrication, limited device material to transmit acoustic power efficiently
Ion concentration polarization	Electric field	Size, electrophoretic mobility	100–500	-	0.0005	Low voltage, no need for internal electrode	Low resolution on small size particles, low throughput
Electrohydro-dynamic vortices	Traveling waves, ohmic heating	Size, charge	200	~100	0.000033	High separation efficiency	Complex fabrication of microelectrode, low throughput
Deterministic lateral displacement	Laminar flow stream	Size, deformability	190–2000	~100	0.00001	Controllable cutoff size, simple and efficient, high separation efficiency (20 nm resolution)	Very low throughput, precise fabrication required, pillar clogging is possible
Hydrodyna-mic filtration	Hydrodynamic sieving	Size	100–1000	-	0.001	Simple, high separation efficiency, medium throughput	Prone to clogging
Spiral microfluidics	Dean vortices	Size, shape	590–7320	95	0.010	Very high separation efficiency, simple	Prone to particle-particle interactions and diffusion disruption
Inertial microfluidics	Shear and wall lift	Size, shape	590–1980	-	-	Very high throughput, separation efficiency, simple	Prone to particle–particle interactions and diffusion disruption
Electrostatic sieving	Electric double-layer force	Size, charge	19–50	97	0.0006	Very high separation efficiency, controllable cut-off size	Separation only possible in low ionic strength conditions, low throughput
Bacterial chemotaxis	Chemotaxis, diffusion, and bacterial motility	Selective adhesion on bacteria	320–390	81	0.000013	Simple, low cost	Requires antibody conjugation for selective adhesion to bacteria, very low throughput, and relatively medium separation efficiency

## 5. Further Application and Processes of Microfluidic Approaches

### 5.1. Purification Strategies

To ensure the high quality of final nanomedicines produced microfluidically, optimization and tailoring of the final purification strategy are required. The classic purification techniques are ultracentrifugation, electrophoresis, chromatography, filtration, size-selective precipitation, and the addition of solvent (Table 4). Despite being effective purification techniques, they depend on the properties of the sample such as purity, density, solubility, and hydrophobicity. Purification methods ideally need to be continuous to combine with microfluidic manufacture and be able to work efficiently with minimal sample volume; utilizing microfluidic separation methods is likely to guarantee a continuous separation, at low costs, which are especially suitable for products with reduced dimensions such as nanoparticles [282]. The parameters that play a role in the successful separation of nanometer samples are size, diffusion, conformational structure, surface forces, pH, and buffers. Surface forces decrease with decreasing particle size, while Brownian movement increases, making separation more complex. Thus, designing devices with internal microchannel structures such as membranes and obstacles such as pillars or pores that can separate particles according to their size by exploiting phenomena such as sieving or laminar flow are typically utilized [248]. The costs for the realization of these devices at nanoscale increase considerably, and it must be added that the separation process is made even more complex by the differences in terms of shapes, structures, and morphological characteristics of the nanoparticle. Nanoparticles have a high surface-to-volume ratio, and this results in a greater aggregation tendency due to the enhanced surface energy. Thus, selection of the appropriate surface microfluidic interactions is critical in the design of a microfluidic purification process and can be achieved by tailoring parameters such as composition, solvents, pH, and temperature. Microfluid separation techniques can be classified into active and passive (Table 4), where external energy sources are used in active techniques; although these techniques guarantee an effective and controlled purification, they are dependent on association with other equipment. Passive techniques are based purely on the device design and action parameters, as it is the hydrodynamic and surface forces that guarantee the separation [248].

### 5.2. Analysis on a Chip and Production with a High Throughput

Integrated and miniaturized analysis on a chip in nanomedicine fabrication can play a crucial role in efficient characterization of different nanomedicines, broadening knowledge about the effect of process parameters and roles of their optimization in final products. On-chip diagnostic and analysis devices can be located at the nanoprecipitation downstream site to enable in situ measurements. Placement of these devices for in situ measurements at other locations through the channel also provides information about the temporal and spatial characteristics of the reaction process [283,284]. An example of this is a microfluidic system based on electrowetting on dielectric coupled to a silicon nanowire-based surface-assisted laser ionization–desorption interface applicable in analysis (mass spectrometry) of small biomolecules [285]. Here, analytes transfer was attained on particular locations on the surface-assisted laser desorption–ionization interface, and their subsequent mass spectrometry evaluations without the application of an organic matrix were performed. To do so, a device comprising a patterned interface of superhydrophilic/superhydrophobic silicon nanowire and a microfluidic system was developed. For the analyte displacement (droplets containing analytes) inside the superhydrophilic patterns via an electrowetting actuation the microfluidic system was served. The nanopatterned silicon interface acted as an inorganic target for the dried analyte matrix-free mass spectrometry analysis. It was demonstrated that the evaluation of compounds with a low molecular weight (700 *m*/*z*) could be attained with a very high sensitivity (down to 10 fmol·μL^−1^) [285]. Furthermore, a tumor-on-a-chip model based on a microfluidic approach was designed for evaluating efficacy and targeting capability of multifunctional liposomes for cancer therapy [286]. The device contained three groups of hemispheric wells with various sizes for the formation of tumor spheroids and assessing of liposomes under a controlled flow regime. There was a good conformity between the tumor targeting capability of fluorescent liposomes during the test in the tumor-on-a-chip model and in in vivo mouse models. In comparison with 3D tumor spheroid models and 2D cell monolayers, the anticancer efficacy evaluation of four paclitaxel-loaded liposome formulations revealed that the developed microfluidic model could better predict the anticancer efficacy (in vivo) of targeted liposomes. Lastly, to study the correlation between treatment efficacy and flow rates, the cytotoxicity of PTX-loaded formulations was evaluated under three flow rates (i.e., 0.25, 1, and 4 µL·min^−1^). The highest cytotoxicity or the lowest spheroid viability using the smallest flow rate was 43.7% and the viability of the tumor spheroid was increased to 60.9% and 69.5% by increasing the flow rate to 1 and 4 µL·min^−1^, respectively, revealing reduced cytotoxicity. The growth curve of the tumor spheroid also showed that lower flow rates resulted in better capability of tumor inhibition. At a flow rate of 0.25 µL·min^−1^, the PTX-loaded liposomes could obviously reduce the volume of tumor spheroids, while the tumor suppression effect was much weaker at the highest flow rate. To further study the influence of the flow rates on the efficacy of tumor inhibitions, the liposome accumulation at the spheroids was evaluated under various flow rates. Quantitative and qualitative results all depicted that higher flow rates resulted in a lower uptake efficiency, showing a reduced liposome accumulation in the tumor spheroid, which might clarify the weaker effect of the tumor suppression at high flow rates. This study demonstrated that the tumor-on-a-chip model could provide a convenient and feasible platform for reliable and rapid cancer drug study [286].

### 5.3. Scale-Up Manufacture

Microreactor application for nanomaterial production on an industrial scale relies extensively on reactor parallelization, where each unit individually process only a small part of a total reaction volume. Scaling up the size of a microfluidic device represents an obvious rise in the used liquid volumetric flow rate that is streamed along the microreactor according to *Q = U × A* (*Q*, *U*, and *A* are the volumetric flow rate, average fluid velocity, microchannel cumulative cross-sectional area, respectively) [154]. A scaling up scheme generally includes the cumulative flow enhancement using the microchannel cross-section via increasing the microreactor numbers. Generally, a considerable increase in average velocity of the fluid leads to unwanted drops in pressure over the microchannel. Commonly, a scaling up strategy has three levels: (i) increasing the channel numbers providing channels identically in the same lamina, (ii) increasing the layer numbers, which create multiple layers with arrays of the channel, and (iii) increasing the device numbers by employing devices with identical structure and function linked in parallel. To obtain an effective microfluidic device with the ability of scaling up the production of nanomaterials, the flow rate must be the same in all the arrayed microchannels. As aforementioned, the flow rate throughout the channel is a key parameter in controlling the characteristics affecting reactions (e.g., mass and heat transfer, particle residence time) and, subsequently, the prepared nanomaterials [1,138,206]. Heterogeneity and large particle size can be improved by enhancing the FRR using HFF devices, but this will also decrease the concentration of LNPs in the product; thus, HFF devices may not be a suitable choice for LNP mass production using a lipid solution with a high concentration [245,265,287,288]. Pumps for each individual channel can satisfy the necessity of a uniform flow distribution across the arrayed channels but increases manufacturing costs. A more practical approach is distributing the flow from a main joint reservoir along microchannels to a common reservoir for the prepared product. However, achieving a uniform flow rate for all the distributed flows in each channel is not an easy task [289]. Comparing the conventional extrusion method with microfluidics for liposome (sphingomyelin/cholesterol) production and scale-up [290] showed that an increase in mixing ratio and higher flow rate ratio led to smaller liposomes, resulting in liposomes with a lipid concentration and size appropriate for clinical translation. The cellular efficacy data indicated that the vinblastine-*N*-oxide-loaded liposomes prepared using the microfluidic method and microfluidically prepared/freeze-dried vinblastine-*N*-oxide-loaded liposomes achieved similar efficacy to vinblastine-*N*-oxide-loaded liposomes prepared using the extrusion method. The maximum tolerated dose and pharmacokinetic studies further demonstrated that there was no difference between the in vivo properties of the vinblastine-*N*-oxide-loaded liposome manufactured using the extrusion or microfluidics methods [290]. In another study, PEGylated liposomal doxorubicin was prepared microfluidically followed by tangential flow filtration allowed for scalable production [291] of liposomes with critical quality attributes comparable to those of Caelyx^®^/Doxil^®^. High encapsulation efficiencies (EE% ≥ 90) were attained for all three assessed drugs (doxorubicin, acridine orange, and vincristine). Thus, these microfluidically doxorubicin-loaded liposomes demonstrated comparable physicochemical behavior and pharmaceutical quality criteria correlation to Doxil^®^/Caelyx^®^ in terms of liposomal size, zeta potential, size distribution, drug loading, product sterilization, particle stability, and drug release, while the control of CQAs was possible in a scale-independent manner [291].

**Figure 6:**
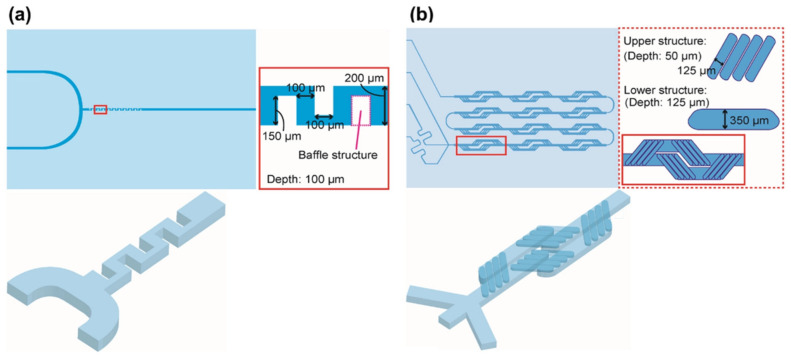
**Schematic of (a) iLiNP (two inlets) and (b) micromixer device**. Modified from [299] and used under the Creative Commons license permission (CC BY 4.0). Copyright 2022, American Chemical Society. All rights reserved.

The lipid concentration typically used for microfluidically prepared lipid-based nanomedicines is low; for example, in the case of liposomes produced with an FRR of 10 or 30, the lipid concentration varied between 0.16 and 0.45 mM, respectively [168]. Even at studies where the lipid concentration was higher (0.1–2 mM depending on FRR) using MHF mixers, the concentration was far lower than the clinical liposomal formulation (5–25 mM) [35]. One way to overcome this is by feeding a highly concentrated lipid solution into microfluidic devices to boost productivity. The advantage of using a solution with high lipid concentrations is the capability of fabricating concentrated LNPs in a short run time, which allows for dilution to obtain a sample with desired concentration. The feasibility of mass production of highly concentrated LNPs is more desirable than their concentration using tangential flow filtration or ultracentrifugation. This can also prevent alterations in the particle characteristics of the LNPs within concentration steps. However, the use of high lipid concentrations is at the expense of control of particle size (polydispersity) and results in larger particle sizes [86,292,293,294,295,296,297,298]. Matsuura-Sawada et al. studied the controllability of LNP production with low-to-high lipid concentrations using iLiNP baffle mixes (microchannels with a height and width of 100 and 200 µm, respectively) [299]. The interval, length, and width of each baffle were 100, 100, and 150 μm (a total of 20 baffles) (Figure 6) [299], and the FRR was fixed to 3. They fixed the FRR of the lipid phase to the aqueous phase at 3. At a lipid (POPC) concentration of 10 mg/mL, LNPs smaller than 100 nm were produced; however, when the concentration increased 10-fold, even at high flow rate conditions, the LNPs size was in the range of 130–140 nm (PDI < 0.2) [299].

The lipid concentration effects on the size of LNPs produced using iLiNP at a TFR of 1000 μL/min (POPC concentration 10–50 mg/mL) resulted in LNPs < 100 nm. Applying the same conditions in the micromixer device (Figure 6) led to LNPs at least 1.4 times bigger than those fabricated by iLiNP [299]. It was found that iLiNP at a TFR of 500 μL/min (FRRs of 3 and 9) could attain a complete mixing state within 3 ms [269], where, at a TFR of 500 μL/min (FRR of 1), approximately 192 ms was required for the micromixer device to attain a complete mixing state [300]. In the range of 10–50 mg/mL for POPC, this was comparable to the differences in the size of LNPs [299]. The LNPs manufactured at a concentration of 100 mg/mL POPC using iLiNP represented a roughly similar size reduction trend to those fabricated using the micromixer device. By increasing TFR from 500 to 1000 μL/min, the size of the produced LNPs using iLiNP remained at 130 nm, but LNPs prepared using the micromixer device showed a reduction in size upon increasing TFR. These results depicted that the threshold concentration of POPC is between 50 and 100 mg/mL for various size decrement tendencies using the micromixer and iLiNP devices [299]. It also showed that higher concentration of LNPs can be manufactured using iLiNP per unit volume, which suggests that microfluidic devices have the feasibility for mass production of size-controlled LNPs.

Acoustically (ultrasound) driven microfluidic micromixers can potentially allow for feeding higher lipid concentrations; however, this has still not been demonstrated [301]. The acoustic microstreaming reinforced by integrating sharp edges and bubbles in the devices can trigger higher throughput of liposomes with lower polydispersity, along with a controlled size at lower FRR, which can enable higher lipid concentrations, avoiding large nanoparticle aggregates and clogging of the channel [302]. However, the vibrational amplitude of sharp edges rapidly diminished as the flow rate increased, limiting the throughput.

Thus, to prepare lipid nanomedicines for drug delivery, micromixers may generally be a better choice because of their relatively high encapsulation efficiency, easy usability, and full use of encapsulated materials. The throughput of micromixers was exponentially scaled up by incorporating an array of numerous mixing channels that operate simultaneously [303]. In fact, commercial devices based on micromixers such as NanoAssemblr™ platforms have been universally used in many studies to prepare lipid particles for different applications, such as CRISPR/Cas9 genome editing for cancer therapy [304] and in utero mRNA delivery for monogenic fetal diseases [305] Notably, COVID-19 vaccine nanoparticles manufactured by Pfizer were scaled up using parallel microfluidic mixers [295,306].

## 6. Future Perspectives and Challenges

Microfluidic approaches enable the continuous manufacture of lipid-based nanomedicines for preclinical and clinical administration with controlled properties, efficacy, and safety profiles, as the latter are linked to particle properties. Developing continuous and scalable approaches to the manufacture of nanomedicines will allow for the translation of novel technologies and enhance the likelihood of uptake of these technologies by the pharmaceutical industry. Knowledge transfer among academic institutions, industrial partners, and clinicians creates the ideal environment for innovation needed to enable nanomedicine translation to overcome societal challenges as shown in the case of COVID-19 vaccines. Current advances in 3D printing technologies enable facile generation of tailored systems for pilot studies that can make initial optimization a facile and cost-effective process compared to planar micromachining approaches such as soft lithography. Continuous flow microfluidic fabrication methods are amenable to scale-up processes, while digital microfluidics with liquid marbles and droplets can address the issue of cumbersome-to-manufacture constructs.

## Figures and Tables

**Table 1:** Summary of main components, characteristics, methods of manufacture, and advantages and disadvantages of lipid-based nanosystems.

System	Components	Diameter (nm)/Shape	Manufacturing	Pros	Cons	Refs.
Transferosomes	Edge activators, phospholipids	Less than 300/spherical bilayer	Vortexing, sonication, rotary film, or reverse-phase evaporations	Good stability, higher penetration	Susceptible to oxidative degradation	[36,90]
Liposomes	Cholesterol, phospholipids, essential oils	10–1000/spherical bilayers	Solvent dispersion, mechanical dispersion, detergent removal	Controlled release, drug protection, solubility improvement for hydrophobic drugs, high biodistribution and bioavailability	Rigid structure, limited penetration across the stratum corneum	[28,29,32,33,62,90]
Nanostructured lipid carriers (NLCs)	Liquid and solid lipids, surfactants	50–1000/spherical single layer	Sonication, micro emulsification, high-pressure homogenization	High cell uptake, appropriate protection of therapeutics in acidic pH, biodegradable and biocompatible, simplicity of drug entrapment, long shelf-life, more sustainable drug dissolution, high payload, reduced loss of drug during storage	Solid/liquid lipid ratio optimization difficulties	[52,53,91,92,93]
Solid lipid nanoparticles (SLNs)	Surfactants, solid lipids	50–1000/spherical single layer	Sonication, micro emulsification, high-pressure homogenization	High cell uptake, appropriate protection of therapeutics in acidic pH, biodegradable and biocompatible ingredients, simplicity of drug entrapment, long shelf-life	Gelling tendency	[50,51,52,91,92,93,94,95,96,97,98]
Ethosomes	Phospholipids, edge activator, high concentration of a low-molecular-weight alcohol (ethanol ≤45% *w*/*w*), water	<200 or 300/spherical with diverse lamellarities	Solvent dispersion, ethanol injection–sonication, thin-film hydration, reverse-phase evaporation, transmembrane pH gradient, extrusion, and sonication	Increased efficacy and therapeutic index, reduce toxicity of API, improved permeation, entrapment of lipophilic, hydrophilic, and amphiphilic agents, simplicity of manufacturing, noninvasive, better solubility and stability, selective passive targeting	Low production yield, only for potent drugs, skin dermatitis or irritation may occur, drug leakage during transfer from organic to water media	[99,100]
Cochleates	Phospholipid–cation precipitates (formed by a continuous, solid, lipid bilayer sheet rolled up in a spiral)	100–1000/cylindrical shape	Trapping method (a bridging agent is mixed with an aqueous lipid suspension), hydrogel method, dialysis, emulsification–lyophilization, microfluidic method, solvent injection	Nonimmunogenic, noninflammatory, and nontoxic, a stable structure due to their tightly packed nature, less susceptible to oxidation of both the encapsulated drug and the phospholipids, sustained release is achievable, enhanced shelf-life, improvements in oral bioavailability of drugs	Aggregation on storage, high production cost	[56,101]

**Table 2:** Summary of liposome preparation methods and their suitability for continuous manufacturing Reprinted/adapted with permission from [117] and used under the Creative Commons license permission (CC BY 4.0). Copyright 2018, John Wiley & Sons, Inc. All rights reserved.

Method	Mechanism	Suitability for Continuous Manufacturing
Bangham	Rehydration of thin lipid film	Not practical—needs continuous dehydration/rehydration steps
Sonication	Sonication of aqueous lipid suspensions	Requires small-scale batch operation to ensure sonication efficiency
Reverse-phase evaporation	Aqueous phase added to organic phase and evaporated to form liposomes	Very complex to regulate continuous solvent evaporation, sterile boundary hard to establish
Detergent depletion	Liposomes forms through detergent–lipid interaction	Slow process with difficult-to-establish sterile boundary, detergent use generally disadvantageous
Microfluidic channel	Intersection of lipid and API solutions in micromixers	Continuous but small/medium scale that can be upscaled in parallel
High-pressure homogenization	Liposome formation through high-pressure mixing	Very high pressures required, difficulty in sterilizing equipment
Heating	Heating of lipid aqueous/glycerol solution to form liposomes	Hydration step and high temperatures make continuous production impractical
Supercritical fluid methods	Use of supercritical fluids as solvent for lipids instead	High pressures required for feed vessels make resupply/continuous operation impractical
Dense gas	Use of dense gas as solvent for lipids	High pressures required for feed vessels make resupply/continuous operation impractical
Dual asymmetric centrifugation	Mechanical turbulence and cavitation	Only for batch sizes ~1 g or less
Ethanol/ether injection	Precipitation of liposome from organic phase into aqueous	Simple process with inherently continuous liposome formation step
Crossflow	In-line precipitation of liposome from organic phase into aqueous	Simple process with inherently continuous liposome formation step

## Data Availability

Not applicable.

## Supplementary Materials

The following supporting information can be downloaded at https://www.mdpi.com/article/10.3390/pharmaceutics14091940/s1: Table S1. Lipid-based formulations in clinical trials.

## References

[1] Kara A., Vassiliadou A., Ongoren B., Keeble W., Hing R., Lalatsa A., Serrano D.R. (2021). Engineering 3D Printed Microfluidic Chips for the Fabrication of Nanomedicines. Pharmaceutics.

[2] Abedinoghli D., Charkhpour M., Osouli-Bostanabad K., Selselehjonban S., Emami S., Barzegar-Jalali M., Adibkia K. (2018). Electrosprayed Nanosystems of Carbamazepine—PVP K30 for Enhancing Its Pharmacologic Effects. Iran. J. Pharm. Res..

[3] Barenholz Y. (2012). Doxil^®^—The first FDA-approved nano-drug: Lessons learned. J. Control. Release.

[4] Specification P.A. (2007). Terminology for Nanomaterials.

[5] Bosetti R., Jones S.L. (2019). Cost–effectiveness of nanomedicine: Estimating the real size of nano-costs. Nanomedicine.

[6] Peptide Therapeutics Market (2020). Reports and Data. https://www.reportsanddata.com/report-detail/peptide-therapeutics-market.

[7] Bobo D., Robinson K.J., Islam J., Thurecht K.J., Corrie S.R. (2016). Nanoparticle-Based Medicines: A Review of FDA-Approved Materials and Clinical Trials to Date. Pharm. Res..

[8] Germain M., Caputo F., Metcalfe S., Tosi G., Spring K., Åslund A.K.O., Pottier A., Schiffelers R., Ceccaldi A., Schmid R. (2020). Delivering the power of nanomedicine to patients today. J. Control. Release.

[9] Anselmo A.C., Mitragotri S. (2019). Nanoparticles in the clinic: An update. Bioeng. Transl. Med..

[10] Forssen E.A. (1997). The design and development of DaunoXome® for solid tumor targeting in vivo. Adv. Drug Deliv. Rev..

[11] Boswell G.W., Buell D., Bekersky I. (1998). AmBisome (Liposomal Amphotericin B): A Comparative Review. J. Clin. Pharmacol..

[12] Bulbake U., Doppalapudi S., Kommineni N., Khan W. (2017). Liposomal Formulations in Clinical Use: An Updated Review. Pharmaceutics.

[13] Silverman J.A., Deitcher S.R. (2013). Marqibo^®^ (vincristine sulfate liposome injection) improves the pharmacokinetics and pharmacodynamics of vincristine. Cancer Chemother. Pharmacol..

[14] Liu X., Jiang J., Chan R., Ji Y., Lu J., Liao Y.-P., Okene M., Lin J., Lin P., Chang C.H. (2019). Improved Efficacy and Reduced Toxicity Using a Custom-Designed Irinotecan-Delivering Silicasome for Orthotopic Colon Cancer. ACS Nano.

[15] Akinc A., Maier M.A., Manoharan M., Fitzgerald K., Jayaraman M., Barros S., Ansell S., Du X., Hope M.J., Madden T.D. (2019). The Onpattro story and the clinical translation of nanomedicines containing nucleic acid-based drugs. Nat. Nanotechnol..

[16] Choi Y.H., Han H.-K. (2018). Nanomedicines: Current status and future perspectives in aspect of drug delivery and pharmacokinetics. J. Pharm. Investig..

[17] Martins J.P., das Neves J., de la Fuente M., Celia C., Florindo H., Günday-Türeli N., Popat A., Santos J.L., Sousa F., Schmid R. (2020). The solid progress of nanomedicine. Drug Deliv. Transl. Res..

[18] Thi T.T.H., Suys E.J.A., Lee J.S., Nguyen D.H., Park K.D., Truong N.P. (2021). Lipid-Based Nanoparticles in the Clinic and Clinical Trials: From Cancer Nanomedicine to COVID-19 Vaccines. Vaccines.

[19] Mitchell M.J., Billingsley M.M., Haley R.M., Wechsler M.E., Peppas N.A., Langer R. (2021). Engineering precision nanoparticles for drug delivery. Nat. Rev. Drug Discov..

[20] Shin M.D., Shukla S., Chung Y.H., Beiss V., Chan S.K., Ortega-Rivera O.A., Wirth D.M., Chen A., Sack M., Pokorski J.K. (2020). COVID-19 vaccine development and a potential nanomaterial path forward. Nat. Nanotechnol..

[21] Soares S., Sousa J., Pais A., Vitorino C. (2018). Nanomedicine: Principles, Properties, and Regulatory Issues. Front. Chem..

[22] Selselehjonban S., Garjani A., Osouli-Bostanabad K., Tanhaei A., Emami S., Adibkia K., Barzegar-Jalali M. (2019). Physicochemical and pharmacological evaluation of carvedilol-eudragit(^®^) RS100 electrosprayed nanostructures. Iran. J. Basic Med. Sci..

[23] Kim B.Y.S., Rutka J.T., Chan W.C.W. (2010). Nanomedicine. N. Engl. J. Med..

[24] Khurana A., Allawadhi P., Khurana I., Allwadhi S., Weiskirchen R., Banothu A.K., Chhabra D., Joshi K., Bharani K.K. (2021). Role of nanotechnology behind the success of mRNA vaccines for COVID-19. Nano Today.

[25] Ahn J., Ko J., Lee S., Yu J., Kim Y., Jeon N.L. (2018). Microfluidics in nanoparticle drug delivery; From synthesis to pre-clinical screening. Adv. Drug Deliv. Rev..

[26] Fornaguera C., García-Celma M.J. (2017). Personalized Nanomedicine: A Revolution at the Nanoscale. J. Pers. Med..

[27] Tapeinos C., Battaglini M., Ciofani G. (2017). Advances in the design of solid lipid nanoparticles and nanostructured lipid carriers for targeting brain diseases. J. Control. Release.

[28] Fonseca-Santos B., Gremião M.P.D., Chorilli M. (2015). Nanotechnology-based drug delivery systems for the treatment of Alzheimer’s disease. Int. J. Nanomed..

[29] Sercombe L., Veerati T., Moheimani F., Wu S.Y., Sood A.K., Hua S. (2015). Advances and Challenges of Liposome Assisted Drug Delivery. Front. Pharmacol..

[30] Fenton O.S., Olafson K.N., Pillai P.S., Mitchell M.J., Langer R. (2018). Advances in Biomaterials for Drug Delivery. Adv. Mater..

[31] Sarfraz M., Afzal A., Yang T., Gai Y., Raza S.M., Khan M.W., Cheng Y., Ma X., Xiang G. (2018). Development of Dual Drug Loaded Nanosized Liposomal Formulation by A Reengineered Ethanolic Injection Method and Its Pre-Clinical Pharmacokinetic Studies. Pharmaceutics.

[32] Sedighi M., Sieber S., Rahimi F., Shahbazi M.-A., Rezayan A.H., Huwyler J., Witzigmann D. (2019). Rapid optimization of liposome characteristics using a combined microfluidics and design-of-experiment approach. Drug Deliv. Transl. Res..

[33] Lalatsa A., Schätzlein A.G., Uchegbu I.F. (2019). Drug delivery across the blood-brain barrier. Comprehensive Biotechnology.

[34] Fernández-García R., Lalatsa A., Statts L., Bolás-Fernández F., Ballesteros M.P., Serrano D.R. (2020). Transferosomes as nanocarriers for drugs across the skin: Quality by design from lab to industrial scale. Int J. Pharm.

[35] Rai S., Pandey V., Rai G. (2017). Transfersomes as versatile and flexible nano-vesicular carriers in skin cancer therapy: The state of the art. Nano Rev. Exp..

[36] Opatha S.A.T., Titapiwatanakun V., Chutoprapat R. (2020). Transfersomes: A Promising Nanoencapsulation Technique for Transdermal Drug Delivery. Pharmaceutics.

[37] Naik U.S. (2013). The Synthesis and Characterisation of Novel Ultra-Flexible Lipidic Vesicles Using Propanol. Ph.D. Thesis.

[38] Rane B.R., Gujarathi N.A., Keservani R.K., Sharma A.K., Kesharwani R.K. (2017). Transfersomes and Protransfersome: Ultradeformable Vesicular System. Novel Approaches for Drug Delivery.

[39] Rajan R., Jose S., Biju Mukund V., Vasudevan D. (2011). Transferosomes—A vesicular transdermal delivery system for enhanced drug permeation. J. Adv. Pharm. Technol. Res..

[40] Jangdey M.S., Gupta A., Saraf S., Saraf S. (2017). Development and optimization of apigenin-loaded transfersomal system for skin cancer delivery: In vitro evaluation. Artif. Cells Nanomed. Biotechnol..

[41] Touitou E. (1996). Compositions for Applying Active Substances to or through the Skin. U.S. Patent.

[42] Touitou E., Dayan N., Bergelson L., Godin B., Eliaz M. (2000). Ethosomes—Novel vesicular carriers for enhanced delivery: Characterization and skin penetration properties. J. Control. Release.

[43] Touitou E. (1998). Composition for Applying Active Substances to or through the Skin. U.S. Patent.

[44] Saifi Z., Rizwanullah M., Mir S.R., Amin S. (2020). Bilosomes nanocarriers for improved oral bioavailability of acyclovir: A complete characterization through in vitro, ex-vivo and in vivo assessment. J. Drug Deliv. Sci. Technol..

[45] Conacher M., Alexander J., Brewer J.M. (2001). Oral immunisation with peptide and protein antigens by formulation in lipid vesicles incorporating bile salts (bilosomes). Vaccine.

[46] Pavlović N., Goločorbin-Kon S., Ðanić M., Stanimirov B., Al-Salami H., Stankov K., Mikov M. (2018). Bile Acids and Their Derivatives as Potential Modifiers of Drug Release and Pharmacokinetic Profiles. Front. Pharmacol..

[47] Niu M., Lu Y., Hovgaard L., Guan P., Tan Y., Lian R., Qi J., Wu W. (2012). Hypoglycemic activity and oral bioavailability of insulin-loaded liposomes containing bile salts in rats: The effect of cholate type, particle size and administered dose. Eur. J. Pharm. Biopharm..

[48] Aburahma M.H. (2016). Bile salts-containing vesicles: Promising pharmaceutical carriers for oral delivery of poorly water-soluble drugs and peptide/protein-based therapeutics or vaccines. Drug Deliv..

[49] Müller R.H., Mäder K., Gohla S. (2000). Solid lipid nanoparticles (SLN) for controlled drug delivery—A review of the state of the art. Eur. J. Pharm. Biopharm..

[50] Patel S., Ryals R.C., Weller K.K., Pennesi M.E., Sahay G. (2019). Lipid nanoparticles for delivery of messenger RNA to the back of the eye. J. Control. Release.

[51] Vhora I., Lalani R., Bhatt P., Patil S., Misra A. (2019). Lipid-nucleic acid nanoparticles of novel ionizable lipids for systemic BMP-9 gene delivery to bone-marrow mesenchymal stem cells for osteoinduction. Int. J. Pharm..

[52] Duan Y., Dhar A., Patel C., Khimani M., Neogi S., Sharma P., Kumar N.S., Vekariya R.L. (2020). A brief review on solid lipid nanoparticles: Part and parcel of contemporary drug delivery systems. RSC Adv..

[53] Laffleur F., Keckeis V. (2020). Advances in drug delivery systems: Work in progress still needed?. Int. J. Pharm. X.

[54] Zarif L., Graybill J.R., Perlin D., Mannino R.J. (2000). Cochleates: New Lipid-Based Drug Delivery System. J. Liposome Res..

[55] Zarif L. (2002). Elongated supramolecular assemblies in drug delivery. J. Control. Release.

[56] Shende P., Khair R., Gaud R.S. (2019). Nanostructured cochleates: A multi-layered platform for cellular transportation of therapeutics. Drug Dev. Ind. Pharm..

[57] Talke S., Salunkhe K., Chavan M. (2018). A Review on nanocochleates novel approach for drug delivery. World J. Pharm. Pharm. Sci..

[58] Shi J., Kantoff P.W., Wooster R., Farokhzad O.C. (2017). Cancer nanomedicine: Progress, challenges and opportunities. Nat. Rev. Cancer.

[59] Xu X., Ho W., Zhang X., Bertrand N., Farokhzad O. (2015). Cancer nanomedicine: From targeted delivery to combination therapy. Trends Mol. Med..

[60] Yingchoncharoen P., Kalinowski D.S., Richardson D.R. (2016). Lipid-Based Drug Delivery Systems in Cancer Therapy: What Is Available and What Is Yet to Come. Pharmacol. Rev..

[61] Ventola C.L. (2017). Progress in Nanomedicine: Approved and Investigational Nanodrugs. Pharm. Ther..

[62] Shah S., Dhawan V., Holm R., Nagarsenker M.S., Perrie Y. (2020). Liposomes: Advancements and innovation in the manufacturing process. Adv. Drug Deliv. Rev..

[63] Fan Y., Marioli M., Zhang K. (2021). Analytical characterization of liposomes and other lipid nanoparticles for drug delivery. J. Pharm. Biomed. Anal..

[64] Lamichhane N., Udayakumar T.S., D’Souza W.D., Simone II C.B., Raghavan S.R., Polf J., Mahmood J. (2018). Liposomes: Clinical Applications and Potential for Image-Guided Drug Delivery. Molecules.

[65] Spectrum Pharmaceuticals, Inc. Topotecan Liposomes Injection for Small Cell Lung Cancer (SCLC), Ovarian Cancer and Other Advanced Solid Tumors. https://clinicaltrials.gov/ct2/show/NCT00765973.

[66] Swiss Group for Clinical Cancer Research TLD-1, a Novel Liposomal Doxorubicin, in Patients with Advanced Solid Tumors. https://clinicaltrials.gov/ct2/show/NCT03387917.

[67] Mebiopharm Co., Ltd. Safety Study of MBP-426 (Liposomal Oxaliplatin Suspension for Injection) to Treat Advanced or Metastatic Solid Tumors. https://clinicaltrials.gov/ct2/show/NCT00355888.

[68] Mebiopharm Co., Ltd. Study of MBP-426 in Patients with Second Line Gastric, Gastroesophageal, or Esophageal Adenocarcinoma. https://www.clinicaltrials.gov/ct2/show/NCT00964080.

[69] Munster P., Krop I.E., LoRusso P., Ma C., Siegel B.A., Shields A.F., Molnár I., Wickham T.J., Reynolds J., Campbell K. (2018). Safety and pharmacokinetics of MM-302, a HER2-targeted antibody–liposomal doxorubicin conjugate, in patients with advanced HER2-positive breast cancer: A phase 1 dose-escalation study. Br. J. Cancer.

[70] Celsion Study of ThermoDox with Standardized Radiofrequency Ablation (RFA) for Treatment of Hepatocellular Carcinoma (HCC) (OPTIMA). https://clinicaltrials.gov/ct2/show/NCT02112656.

[71] Mebiopharm Co., Ltd. Active Targeting Drug Delivery System. http://www.mebiopharm.com/english/pro.html.

[72] Yonezawa S., Koide H., Asai T. (2020). Recent advances in siRNA delivery mediated by lipid-based nanoparticles. Adv. Drug Deliv. Rev..

[73] Ely A., Singh P., Smith T.S., Arbuthnot P. (2021). In vitro transcribed mRNA for expression of designer nucleases: Advantages as a novel therapeutic for the management of chronic HBV infection. Adv. Drug Deliv. Rev..

[74] McGoron A.J. (2020). Perspectives on the Future of Nanomedicine to Impact Patients: An Analysis of US Federal Funding and Interventional Clinical Trials. Bioconj. Chem..

[75] ModernaTX, Inc., AstraZeneca Dose Escalation Study of mRNA-2752 for Intratumoral Injection to Participants in Advanced Malignancies. https://clinicaltrials.gov/ct2/show/NCT03739931.

[76] ModernaTX, Inc. Dose Escalation and Efficacy Study of mRNA-2416 for Intratumoral Injection Alone and in Combination with Durvalumab for Participants with Advanced Malignancies. https://clinicaltrials.gov/ct2/show/NCT03323398.

[77] National Cancer Institute (NCI) T4N5 Liposomal Lotion in Preventing The Recurrence of Nonmelanoma Skin Cancer in Patients Who Have Undergone a Kidney Transplant. https://clinicaltrials.gov/ct2/show/NCT00089180.

[78] BioNTech, SE Evaluation of the Safety and Tolerability of i.v. Administration of a Cancer Vaccine in Patients with Advanced Melanoma (Lipo-MERIT). https://clinicaltrials.gov/ct2/show/NCT02410733.

[79] ModernaTX, Inc., Merck Sharp & Dohme Corp An Efficacy Study of Adjuvant Treatment with the Personalized Cancer Vaccine mRNA-4157 and Pembrolizumab in Participants with High-Risk Melanoma (KEYNOTE-942). https://clinicaltrials.gov/ct2/show/NCT03897881.

[80] Ebinger J.E., Fert-Bober J., Printsev I., Wu M., Sun N., Prostko J.C., Frias E.C., Stewart J.L., Van Eyk J.E., Braun J.G. (2021). Antibody responses to the BNT162b2 mRNA vaccine in individuals previously infected with SARS-CoV-2. Nat. Med..

[81] Weiss C., Carriere M., Fusco L., Capua I., Regla-Nava J.A., Pasquali M., Scott J.A., Vitale F., Unal M.A., Mattevi C. (2020). Toward Nanotechnology-Enabled Approaches against the COVID-19 Pandemic. ACS Nano.

[82] Kulkarni J.A., Cullis P.R., van der Meel R. (2018). Lipid Nanoparticles Enabling Gene Therapies: From Concepts to Clinical Utility. Nucleic Acid Ther..

[83] Rudra A., Li J., Shakur R., Bhagchandani S., Langer R. (2020). Trends in Therapeutic Conjugates: Bench to Clinic. Bioconj. Chem..

[84] Van Riel D., de Wit E. (2020). Next-generation vaccine platforms for COVID-19. Nat. Mater..

[85] Ng W.H., Liu X., Mahalingam S. (2020). Development of vaccines for SARS-CoV-2. F1000Res.

[86] Ickenstein L.M., Garidel P. (2019). Lipid-based nanoparticle formulations for small molecules and RNA drugs. Expert Opin. Drug Deliv..

[87] Hu B., Zhong L., Weng Y., Peng L., Huang Y., Zhao Y., Liang X.-J. (2020). Therapeutic siRNA: State of the art. Signal Transduct. Target. Ther..

[88] Anderluzzi G., Schmidt S.T., Cunliffe R., Woods S., Roberts C.W., Veggi D., Ferlenghi I., O’Hagan D.T., Baudner B.C., Perrie Y. (2021). Rational design of adjuvants for subunit vaccines: The format of cationic adjuvants affects the induction of antigen-specific antibody responses. J. Control. Release.

[89] Lee K., Kim S.Y., Seo Y., Kim M.H., Chang J., Lee H. (2020). Adjuvant incorporated lipid nanoparticles for enhanced mRNA-mediated cancer immunotherapy. Biomater. Sci..

[90] Jain S., Tripathi S., Tripathi P.K. (2021). Invasomes: Potential vesicular systems for transdermal delivery of drug molecules. J. Drug Deliv. Sci. Technol..

[91] Nasirizadeh S., Malaekeh-Nikouei B. (2020). Solid lipid nanoparticles and nanostructured lipid carriers in oral cancer drug delivery. J. Drug Deliv. Sci. Technol..

[92] Chacko I.A., Ghate V.M., Dsouza L., Lewis S.A. (2020). Lipid vesicles: A versatile drug delivery platform for dermal and transdermal applications. Colloids Surf. B Biointerfaces.

[93] Xu Y., Michalowski C.B., Beloqui A. (2021). Advances in lipid carriers for drug delivery to the gastrointestinal tract. Curr. Opin. Colloid Interface Sci..

[94] Leung A.K.K., Tam Y.Y.C., Chen S., Hafez I.M., Cullis P.R. (2015). Microfluidic Mixing: A General Method for Encapsulating Macromolecules in Lipid Nanoparticle Systems. J. Phys. Chem. B.

[95] Kulkarni J.A., Witzigmann D., Leung J., Tam Y.Y.C., Cullis P.R. (2019). On the role of helper lipids in lipid nanoparticle formulations of siRNA. Nanoscale.

[96] Cheng X., Lee R.J. (2016). The role of helper lipids in lipid nanoparticles (LNPs) designed for oligonucleotide delivery. Adv. Drug Deliv. Rev..

[97] Cheng Q., Wei T., Farbiak L., Johnson L.T., Dilliard S.A., Siegwart D.J. (2020). Selective organ targeting (SORT) nanoparticles for tissue-specific mRNA delivery and CRISPR–Cas gene editing. Nat. Nanotechnol..

[98] Berraondo P., Martini P.G.V., Avila M.A., Fontanellas A. (2019). Messenger RNA therapy for rare genetic metabolic diseases. Gut.

[99] Razavi H., Janfaza S. (2015). Ethosome: A nanocarrier for transdermal drug delivery. Arch. Adv. Biosci..

[100] Abdulbaqi I.M., Darwis Y., Khan N.A.K., Abou Assi R., Khan A.A. (2016). Ethosomal nanocarriers: The impact of constituents and formulation techniques on ethosomal properties, in vivo studies, and clinical trials. Int. J. Nanomed..

[101] Lipa-Castro A., Legrand F.-X., Barratt G. (2021). Cochleate drug delivery systems: An approach to their characterization. Int. J. Pharm..

[102] World Health Organisation (2021). COVID-19 Vaccine Tracker and Landscape. https://www.who.int/publications/m/item/draft-landscape-of-covid-19-candidate-vaccines.

[103] Fang E., Liu X., Li M., Zhang Z., Song L., Zhu B., Wu X., Liu J., Zhao D., Li Y. (2022). Advances in COVID-19 mRNA vaccine development. Signal Transduct. Target. Ther..

[104] FDA News Release, Food and Drug Administration (2021). FDA Approves First COVID-19 Vaccine. https://www.fda.gov/news-events/press-announcements/fda-approves-first-covid-19-vaccine.

[105] FDA News Release, Food and Drug Administration (2021). FDA Authorizes Pfizer-BioNTech COVID-19 Vaccine for Emergency Use in Children 5 through 11 Years of Age. https://www.fda.gov/news-events/press-announcements/fda-authorizes-pfizer-biontech-covid-19-vaccine-emergency-use-children-5-through-11-years-age.

[106] Koirala A., Joo Y.J., Khatami A., Chiu C., Britton P.N. (2020). Vaccines for COVID-19: The current state of play. Paediatr. Respir. Rev..

[107] Samaridou E., Heyes J., Lutwyche P. (2020). Lipid nanoparticles for nucleic acid delivery: Current perspectives. Adv. Drug Deliv. Rev..

[108] Theobald N. (2020). Emerging vaccine delivery systems for COVID-19: Functionalised silica nanoparticles offer a potentially safe and effective alternative delivery system for DNA/RNA vaccines and may be useful in the hunt for a COVID-19 vaccine. Drug Discov. Today.

[109] Shih H.-I., Wu C.-J., Tu Y.-F., Chi C.-Y. (2020). Fighting COVID-19: A quick review of diagnoses, therapies, and vaccines. Biomed. J..

[110] Nakamura T., Harashima H. (2020). Dawn of lipid nanoparticles in lymph node targeting: Potential in cancer immunotherapy. Adv. Drug Deliv. Rev..

[111] Bangham A.D., Standish M.M., Watkins J.C. (1965). Diffusion of univalent ions across the lamellae of swollen phospholipids. J. Mol. Biol..

[112] Batzri S., Korn E.D. (1973). Single bilayer liposomes prepared without sonication. Biochim. Biophys. Acta.

[113] Zumbuehl O., Weder H.G. (1981). Liposomes of controllable size in the range of 40 to 180 nm by defined dialysis of lipid/detergent mixed micelles. Biochim. Biophys. Acta.

[114] Smith L., Serrano D.R., Mauger M., Bolás-Fernández F., Dea-Ayuela M.A., Lalatsa A. (2018). Orally Bioavailable and Effective Buparvaquone Lipid-Based Nanomedicines for Visceral Leishmaniasis. Mol. Pharm..

[115] Fernández- García R., Statts L., de Jesus J.A., Dea-Ayuela M.A., Bautista L., Simão R., Bolás-Fernández F., Ballesteros M.P., Laurenti M.D., Passero L.F.D. (2020). Ultradeformable Lipid Vesicles Localize Amphotericin B in the Dermis for the Treatment of Infectious Skin Diseases. ACS Infect. Dis..

[116] Colombo S., Beck-Broichsitter M., Bøtker J.P., Malmsten M., Rantanen J., Bohr A. (2018). Transforming nanomedicine manufacturing toward Quality by Design and microfluidics. Adv. Drug Deliv. Rev..

[117] Worsham R.D., Thomas V., Farid S.S. (2019). Potential of Continuous Manufacturing for Liposomal Drug Products. Biotechnol. J..

[118] Maherani B., Arab-Tehrany E., Mozafari R.M., Gaiani C., Linder M. (2011). Liposomes: A Review of Manufacturing Techniques and Targeting Strategies. Curr. Nanosci..

[119] Pandita D., Ahuja A., Lather V., Benjamin B., Dutta T., Velpandian T., Khar R.K. (2011). Development of Lipid-Based Nanoparticles for Enhancing the Oral Bioavailability of Paclitaxel. AAPS PharmSciTech.

[120] Aditya N.P., Patankar S., Madhusudhan B., Murthy R.S.R., Souto E.B. (2010). Arthemeter-loaded lipid nanoparticles produced by modified thin-film hydration: Pharmacokinetics, toxicological and in vivo anti-malarial activity. Eur. J. Pharm. Sci..

[121] Evers M.J.W., Kulkarni J.A., van der Meel R., Cullis P.R., Vader P., Schiffelers R.M. (2018). State-of-the-Art Design and Rapid-Mixing Production Techniques of Lipid Nanoparticles for Nucleic Acid Delivery. Small Methods.

[122] Pick U. (1981). Liposomes with a large trapping capacity prepared by freezing and thawing of sonicated phospholipid mixtures. Arch. Biochem. Biophys..

[123] Obeid M.A., Tate R.J., Mullen A.B., Ferro V.A., Grumezescu A.M. (2018). Chapter 8—Lipid-based nanoparticles for cancer treatment. Lipid Nanocarriers for Drug Targeting.

[124] Richardson J., Caruso F. (2020). Nanomedicine toward 2040. Nano Lett..

[125] Kamb A. (2005). What’s wrong with our cancer models?. Nat. Rev. Drug Discov..

[126] Tyner K.M., Zou P., Yang X., Zhang H., Cruz C.N., Lee S.L. (2015). Product quality for nanomaterials: Current U.S. experience and perspective. WIREs Nanomed. Nanobiotechnol..

[127] Burghelea T., Segre E., Bar-Joseph I., Groisman A., Steinberg V. (2004). Chaotic flow and efficient mixing in a microchannel with a polymer solution. Phys. Rev. E.

[128] Lee C.-Y., Wang W.-T., Liu C.-C., Fu L.-M. (2016). Passive mixers in microfluidic systems: A review. Chem. Eng. J..

[129] Yaralioglu G.G., Wygant I.O., Marentis T.C., Khuri-Yakub B.T. (2004). Ultrasonic Mixing in Microfluidic Channels Using Integrated Transducers. Anal. Chem..

[130] Lee S.L., O’Connor T.F., Yang X., Cruz C.N., Chatterjee S., Madurawe R.D., Moore C.M.V., Yu L.X., Woodcock J. (2015). Modernizing Pharmaceutical Manufacturing: From Batch to Continuous Production. J. Pharm. Innov..

[131] Whitesides G.M. (2006). The origins and the future of microfluidics. Nature.

[132] Tokeshi M., Sato K. (2016). Micro/Nano Devices for Chemical Analysis. Micromachines.

[133] Karnik R., Gu F., Basto P., Cannizzaro C., Dean L., Kyei-Manu W., Langer R., Farokhzad O.C. (2008). Microfluidic Platform for Controlled Synthesis of Polymeric Nanoparticles. Nano Lett..

[134] Donno R., Gennari A., Lallana E., De La Rosa J.M.R., d’Arcy R., Treacher K., Hill K., Ashford M., Tirelli N. (2017). Nanomanufacturing through microfluidic-assisted nanoprecipitation: Advanced analytics and structure-activity relationships. Int. J. Pharm..

[135] Bramosanti M., Chronopoulou L., Grillo F., Valletta A., Palocci C. (2017). Microfluidic-assisted nanoprecipitation of antiviral-loaded polymeric nanoparticles. Colloids Surf. A Physicochem. Eng. Asp..

[136] Jaradat E., Weaver E., Meziane A., Lamprou D.A. (2021). Microfluidics Technology for the Design and Formulation of Nanomedicines. Nanomaterials.

[137] Beebe D.J., Mensing G.A., Walker G.M. (2002). Physics and Applications of Microfluidics in Biology. Annu. Rev. Biomed. Eng..

[138] Nguyen N.-T., Wereley S.T., Shaegh S.A.M. (2019). Fundamentals and Applications of Microfluidics.

[139] Zhang Z., Zhao P., Xiao G., Lin M., Cao X. (2008). Focusing-enhanced mixing in microfluidic channels. Biomicrofluidics.

[140] Kumar V., Paraschivoiu M., Nigam K.D.P. (2011). Single-phase fluid flow and mixing in microchannels. Chem. Eng. Sci..

[141] Yang Z., Matsumoto S., Goto H., Matsumoto M., Maeda R. (2001). Ultrasonic micromixer for microfluidic systems. Sens. Actuators A Phys..

[142] Glasgow I., Aubry N. (2003). Enhancement of microfluidic mixing using time pulsing. Lab Chip.

[143] Turkyilmazoglu M. (2020). Magnetohydrodynamic Moving Liquid Plug Within a Microchannel: Analytical Solutions. J. Biomech. Eng..

[144] Tsai J.-H., Lin L. (2002). Active microfluidic mixer and gas bubble filter driven by thermal bubble micropump. Sens. Actuators A Phys..

[145] Wu Z., Nguyen N.-T. (2005). Convective–diffusive transport in parallel lamination micromixers. Microfluid. Nanofluidics.

[146] Knight J.B., Vishwanath A., Brody J.P., Austin R.H. (1998). Hydrodynamic Focusing on a Silicon Chip: Mixing Nanoliters in Microseconds. Phys. Rev. Lett..

[147] Kamholz A.E., Yager P. (2002). Molecular diffusive scaling laws in pressure-driven microfluidic channels: Deviation from one-dimensional Einstein approximations. Sens. Actuators B Chem..

[148] Johnson T.J., Ross D., Locascio L.E. (2002). Rapid Microfluidic Mixing. Anal. Chem..

[149] Stroock A.D., Dertinger S.K.W., Ajdari A., Mezić I., Stone H.A., Whitesides G.M. (2002). Chaotic Mixer for Microchannels. Science.

[150] Günther A., Jhunjhunwala M., Thalmann M., Schmidt M.A., Jensen K.F. (2005). Micromixing of Miscible Liquids in Segmented Gas−Liquid Flow. Langmuir.

[151] Song H., Tice J.D., Ismagilov R.F. (2003). A microfluidic system for controlling reaction networks in time. Angew. Chem..

[152] Capretto L., Cheng W., Hill M., Zhang X., Lin B. (2011). Micromixing Within Microfluidic Devices. Microfluidics: Technologies and Applications.

[153] Osouli-Bostanabad K., Masalehdan T., Kapsa R.M.I., Quigley A., Lalatsa A., Bruggeman K.F., Franks S.J., Williams R.J., Nisbet D.R. (2022). Traction of 3D and 4D Printing in the Healthcare Industry: From Drug Delivery and Analysis to Regenerative Medicine. ACS Biomater. Sci. Eng..

[154] Chang C.-H., Paul B.K., Remcho V.T., Atre S., Hutchison J.E. (2008). Synthesis and post-processing of nanomaterials using microreaction technology. J. Nanopart. Res..

[155] Bertuit E., Neveu S., Abou-Hassan A. (2022). High Temperature Continuous Flow Syntheses of Iron Oxide Nanoflowers Using the Polyol Route in a Multi-Parametric Millifluidic Device. Nanomaterials.

[156] Liu Y., Yang G., Hui Y., Ranaweera S., Zhao C.-X. (2022). Microfluidic Nanoparticles for Drug Delivery. Small.

[157] Abou-Hassan A., Bazzi R., Cabuil V. (2009). Multistep continuous-flow microsynthesis of magnetic and fluorescent γ-Fe_2_O_3_@ SiO_2_ core/shell nanoparticles. Angew. Chem..

[158] Ying Y., Chen G., Zhao Y., Li S., Yuan Q. (2008). A high throughput methodology for continuous preparation of monodispersed nanocrystals in microfluidic reactors. Chem. Eng. J..

[159] Boleininger J., Kurz A., Reuss V., Sönnichsen C. (2006). Microfluidic continuous flow synthesis of rod-shaped gold and silver nanocrystals. Phys. Chem. Chem. Phys..

[160] Ju J., Zeng C., Zhang L., Xu N. (2006). Continuous synthesis of zeolite NaA in a microchannel reactor. Chem. Eng. J..

[161] She Q.M., Liu J.H., Aymonier C., Zhou C.H. (2021). In situ fabrication of layered double hydroxide film immobilizing gold nanoparticles in capillary microreactor for efficient catalytic carbonylation of glycerol. Mol. Catal..

[162] Takagi M., Maki T., Miyahara M., Mae K. (2004). Production of titania nanoparticles by using a new microreactor assembled with same axle dual pipe. Chem. Eng. J..

[163] Liu Z., Lu Y., Yang B., Luo G. (2011). Controllable Preparation of Poly(butyl acrylate) by Suspension Polymerization in a Coaxial Capillary Microreactor. Ind. Eng. Chem. Res..

[164] Flögel O., Codée J.D.C., Seebach D., Seeberger P.H. (2006). Microreactor Synthesis of β-Peptides. Angew. Chem. Int. Ed..

[165] Tan Y.-C., Hettiarachchi K., Siu M., Pan Y.-R., Lee A.P. (2006). Controlled Microfluidic Encapsulation of Cells, Proteins, and Microbeads in Lipid Vesicles. J. Am. Chem. Soc..

[166] Lorenceau E., Utada A.S., Link D.R., Cristobal G., Joanicot M., Weitz D.A. (2005). Generation of Polymerosomes from Double-Emulsions. Langmuir.

[167] Shum H.C., Kim J.-W., Weitz D.A. (2008). Microfluidic Fabrication of Monodisperse Biocompatible and Biodegradable Polymersomes with Controlled Permeability. J. Am. Chem. Soc..

[168] Carugo D., Bottaro E., Owen J., Stride E., Nastruzzi C. (2016). Liposome production by microfluidics: Potential and limiting factors. Sci. Rep..

[169] Ali H.S.M., York P., Blagden N. (2009). Preparation of hydrocortisone nanosuspension through a bottom-up nanoprecipitation technique using microfluidic reactors. Int. J. Pharm..

[170] Edel J.B., Fortt R., deMello J.C., deMello A.J. (2002). Microfluidic routes to the controlled production of nanoparticles. Chem. Commun..

[171] Kastner E., Kaur R., Lowry D., Moghaddam B., Wilkinson A., Perrie Y. (2014). High-throughput manufacturing of size-tuned liposomes by a new microfluidics method using enhanced statistical tools for characterization. Int. J. Pharm..

[172] Aranguren A., Torres C.E., Munoz-Camargo C., Osma J.F., Cruz J.C. (2020). Synthesis of Nanoscale Liposomes via Low-Cost Microfluidic Systems. Micromachines.

[173] Zizzari A., Carbone L., Cesaria M., Bianco M., Perrone E., Rendina F., Arima V. (2021). Continuous flow scalable production of injectable size-monodisperse nanoliposomes in easy-fabrication milli-fluidic reactors. Chem. Eng. Sci..

[174] Shestopalov I., Tice J.D., Ismagilov R.F. (2004). Multi-step synthesis of nanoparticles performed on millisecond time scale in a microfluidic droplet-based system. Lab Chip.

[175] Chan E.M., Alivisatos A.P., Mathies R.A. (2005). High-Temperature Microfluidic Synthesis of CdSe Nanocrystals in Nanoliter Droplets. J. Am. Chem. Soc..

[176] Prakash G., Shokr A., Willemen N., Bashir S.M., Shin S.R., Hassan S. (2022). Microfluidic fabrication of lipid nanoparticles for the delivery of nucleic acids. Adv. Drug Deliv. Rev..

[177] Khan S.A., Günther A., Schmidt M.A., Jensen K.F. (2004). Microfluidic Synthesis of Colloidal Silica. Langmuir.

[178] Zhang S.-H., Shen S.-C., Chen Z., Yun J.-X., Yao K.-J., Chen B.-B., Chen J.-Z. (2008). Preparation of solid lipid nanoparticles in co-flowing microchannels. Chem. Eng. J..

[179] Génot V., Desportes S., Croushore C., Lefèvre J.-P., Pansu R.B., Delaire J.A., von Rohr P.R. (2010). Synthesis of organic nanoparticles in a 3D flow focusing microreactor. Chem. Eng. J..

[180] Jahn A., Vreeland W.N., Gaitan M., Locascio L.E. (2004). Controlled Vesicle Self-Assembly in Microfluidic Channels with Hydrodynamic Focusing. J. Am. Chem. Soc..

[181] Yun J., Zhang S., Shen S., Chen Z., Yao K., Chen J. (2009). Continuous production of solid lipid nanoparticles by liquid flow-focusing and gas displacing method in microchannels. Chem. Eng. Sci..

[182] Gupta R., Fletcher D.F., Haynes B.S. (2010). Taylor Flow in Microchannels: A Review of Experimental and Computational Work. J. Comput. Multiph. Flows.

[183] Tice J.D., Song H., Lyon A.D., Ismagilov R.F. (2003). Formation of Droplets and Mixing in Multiphase Microfluidics at Low Values of the Reynolds and the Capillary Numbers. Langmuir.

[184] Kreutzer M.T., Kapteijn F., Moulijn J.A., Heiszwolf J.J. (2005). Multiphase monolith reactors: Chemical reaction engineering of segmented flow in microchannels. Chem. Eng. Sci..

[185] Tan Z., Lan W., Liu Q., Wang K., Hussain M., Ren M., Geng Z., Zhang L., Luo X., Zhang L. (2019). Kinetically Controlled Self-Assembly of Block Copolymers into Segmented Wormlike Micelles in Microfluidic Chips. Langmuir.

[186] Matosevic S., Paegel B.M. (2011). Stepwise Synthesis of Giant Unilamellar Vesicles on a Microfluidic Assembly Line. J. Am. Chem. Soc..

[187] Matosevic S., Paegel B.M. (2013). Layer-by-layer cell membrane assembly. Nat. Chem..

[188] Erfle P., Riewe J., Bunjes H., Dietzel A. (2017). Optically monitored segmented flow for controlled ultra-fast mixing and nanoparticle precipitation. Microfluid. Nanofluidics.

[189] Riewe J., Erfle P., Melzig S., Kwade A., Dietzel A., Bunjes H. (2020). Antisolvent precipitation of lipid nanoparticles in microfluidic systems—A comparative study. Int. J. Pharm..

[190] Zinoveva S., De Silva R., Louis R.D., Datta P., Kumar C.S., Goettert J., Hormes J. (2007). The wet chemical synthesis of Co nanoparticles in a microreactor system: A time-resolved investigation by X-ray absorption spectroscopy. Nucl. Instrum. Methods Phys. Res. Sect. A: Accel. Spectrometers Detect. Assoc. Equip..

[191] Erfan M., Gnambodoe-Capochichi M., Sabry Y.M., Khalil D., Leprince-Wang Y., Bourouina T. (2021). Spatiotemporal dynamics of nanowire growth in a microfluidic reactor. Microsyst. Nanoeng..

[192] Li J., Šimek H., Ilioae D., Jung N., Braese S., Zappe H., Dittmeyer R., Ladewig B.P. (2021). In Situ Sensors for Flow Reactors—A Review. React. Chem. Eng..

[193] Sounart T.L., Safier P.A., Voigt J.A., Hoyt J., Tallant D.R., Matzke C.M., Michalske T.A. (2007). Spatially-resolved analysis of nanoparticle nucleation and growth in a microfluidic reactor. Lab Chip.

[194] Just J., Coughlan C., Singh S., Ren H., Müller O., Becker P., Unold T., Ryan K.M. (2021). Insights into Nucleation and Growth of Colloidal Quaternary Nanocrystals by Multimodal X-ray Analysis. ACS Nano.

[195] Herbst M. (2021). Microfluidic and X-ray Techniques for Investigations of Nanoparticle Nucleation and Growth.

[196] Maeki M., Fujishima Y., Sato Y., Yasui T., Kaji N., Ishida A., Tani H., Baba Y., Harashima H., Tokeshi M. (2017). Understanding the formation mechanism of lipid nanoparticles in microfluidic devices with chaotic micromixers. PLoS ONE.

[197] Wilms D., Klos J., Frey H. (2008). Microstructured Reactors for Polymer Synthesis: A Renaissance of Continuous Flow Processes for Tailor-Made Macromolecules?. Macromol. Chem. Phys..

[198] Yu W., Chen H., Wu H., Lin P., Xu H., Xie Q., Shi K., Xie G., Chen Y. (2022). Continuous-flow rapid synthesis of wavelength-tunable luminescent lanthanide metal-organic framework nanorods by a microfluidic reactor. J. Alloys Compd..

[199] Nakamura H., Yamaguchi Y., Miyazaki M., Maeda H., Uehara M., Mulvaney P. (2002). Preparation of CdSe nanocrystals in a micro-flow-reactor. Chem. Commun..

[200] Zook J.M., Vreeland W.N. (2010). Effects of temperature, acyl chain length, and flow-rate ratio on liposome formation and size in a microfluidic hydrodynamic focusing device. Soft Matter.

[201] Pradhan P., Guan J., Lu D., Wang P.G., Lee L.J., Lee R.J. (2008). A Facile Microfluidic Method for Production of Liposomes. Anticancer Res..

[202] Miranda I., Souza A., Sousa P., Ribeiro J., Castanheira E.M.S., Lima R., Minas G. (2022). Properties and Applications of PDMS for Biomedical Engineering: A Review. J. Funct. Biomater..

[203] Zhang H., Huang L., Tan M., Zhao S., Liu H., Lu Z., Li J., Liang Z. (2022). Overview of 3D-Printed Silica Glass. Micromachines.

[204] Damodara S., Shahriari S., Wu W.-I., Rezai P., Hsu H.-H., Selvaganapathy R., Li X., Zhou Y. (2021). 1—Materials and methods for microfabrication of microfluidic devices. Microfluidic Devices for Biomedical Applications.

[205] Shubhava, Jayarama A., Kannarpady G.K., Kale S., Prabhu S., Pinto R. (2021). Chemical etching of glasses in hydrofluoric Acid: A brief review. Mater. Today Proc..

[206] Bahrani S., Ghalamfarsa F., Nekoi S., Ghaedi M., Hashemi S.A., Mousavi S.M., Ozkan S.A., Bakirhan N.K., Mollarasouli F. (2022). Chapter 17—Microfluidics technology: Past, present, and future prospects for biomarker diagnostics. The Detection of Biomarkers.

[207] Friend J., Yeo L. (2010). Fabrication of microfluidic devices using polydimethylsiloxane. Biomicrofluidics.

[208] Kim P., Kwon K.W., Park M.C., Lee S.H., Kim S.M., Suh K.Y. (2008). Soft Lithography for Microfluidics: A Review. Biochip J..

[209] Perez-Toralla K., Champ J., Mohamadi M.R., Braun O., Malaquin L., Viovy J.-L., Descroix S. (2013). New non-covalent strategies for stable surface treatment of thermoplastic chips. Lab Chip.

[210] Agha A., Waheed W., Alamoodi N., Mathew B., Alnaimat F., Abu-Nada E., Abderrahmane A., Alazzam A. (2022). A Review of Cyclic Olefin Copolymer Applications in Microfluidics and Microdevices. Macromol. Mater. Eng..

[211] Garcia-Rey S., Nielsen J.B., Nordin G.P., Woolley A.T., Basabe-Desmonts L., Benito-Lopez F. (2022). High-Resolution 3D Printing Fabrication of a Microfluidic Platform for Blood Plasma Separation. Polymers.

[212] Stansbury J.J.W., Idacavage M.J. (2016). 3D printing with polymers: Challenges among expanding options and opportunities. Dent. Mater..

[213] Dizon J.R.C., Espera A.H., Chen Q., Advincula R.C. (2018). Mechanical characterization of 3D-printed polymers. Addit. Manuf..

[214] Schoerpf S., Catel Y., Moszner N., Gorsche C., Liska R. (2019). Enhanced reduction of polymerization-induced shrinkage stress via combination of radical ring opening and addition fragmentation chain transfer. Polym. Chem..

[215] Iedema P.D., Schamböck V., Boonen H., van der Linden M.N., Willemse R. (2019). Photocuring of di-acrylate in presence of oxygen. Chem. Eng. Sci..

[216] Peerzada M., Abbasi S., Lau K.T., Hameed N. (2020). Additive Manufacturing of Epoxy Resins: Materials, Methods, and Latest Trends. Ind. Eng. Chem. Res..

[217] Li S., Sun D., Li A., Cui Y. (2021). Study on curing shrinkage and mechanism of DHOM-modified epoxy-acrylate-based UV-curing 3D printing materials. J. Appl. Polym. Sci..

[218] Mohan D., Sajab M.S., Bakarudin S.B., Roslan R., Kaco H. (2021). 3D Printed Polyurethane Reinforced Graphene Nanoplatelets. Mater. Sci. Forum.

[219] Sun B., Jiang J., Shi N., Xu W. (2016). Application of microfluidics technology in chemical engineering for enhanced safety. Process Saf. Prog..

[220] Zhang H., Anoop K., Huang C., Sadr R., Gupte R., Dai J., Han A. (2022). A circular gradient-width crossflow microfluidic platform for high-efficiency blood plasma separation. Sens. Actuators B Chem..

[221] Nix C., Fillet M., Li X., Yang C., Li P.C.H. (2022). Chapter 10—Microfluidics in three key aspects of the drug-development process: Biomarker discovery, preclinical studies, and drug delivery systems. Multidisciplinary Microfluidic and Nanofluidic Lab-on-a-Chip.

[222] Luo X., Su P., Zhang W., Raston C.L. (2019). Microfluidic Devices in Fabricating Nano or Micromaterials for Biomedical Applications. Adv. Mater. Technol..

[223] Ran R., Wang H., Liu Y., Hui Y., Sun Q., Seth A., Wibowo D., Chen D., Zhao C.-X. (2018). Microfluidic self-assembly of a combinatorial library of single- and dual-ligand liposomes for in vitro and in vivo tumor targeting. Eur. J. Pharm. Biopharm..

[224] Li Y., Lee R.J., Huang X., Li Y., Lv B., Wang T., Qi Y., Hao F., Lu J., Meng Q. (2017). Single-step microfluidic synthesis of transferrin-conjugated lipid nanoparticles for siRNA delivery. Nanomed. Nanotechnol. Biol. Med..

[225] Ran R., Middelberg A.P.J., Zhao C.-X. (2016). Microfluidic synthesis of multifunctional liposomes for tumour targeting. Colloids Surf. B Biointerfaces.

[226] Maeki M., Uno S., Niwa A., Okada Y., Tokeshi M. (2022). Microfluidic technologies and devices for lipid nanoparticle-based RNA delivery. J. Control. Release.

[227] Shepherd S.J., Issadore D., Mitchell M.J. (2021). Microfluidic formulation of nanoparticles for biomedical applications. Biomaterials.

[228] Belliveau N.M., Huft J., Lin P.J.C., Chen S., Leung A.K.K., Leaver T.J., Wild A.W., Lee J.B., Taylor R.J., Tam Y.K. (2012). Microfluidic Synthesis of Highly Potent Limit-size Lipid Nanoparticles for In Vivo Delivery of siRNA. Mol. Ther. Nucleic Acids.

[229] Zhigaltsev I.V., Belliveau N., Hafez I., Leung A.K.K., Huft J., Hansen C., Cullis P.R. (2012). Bottom-Up Design and Synthesis of Limit Size Lipid Nanoparticle Systems with Aqueous and Triglyceride Cores Using Millisecond Microfluidic Mixing. Langmuir.

[230] Maeki M., Saito T., Sato Y., Yasui T., Kaji N., Ishida A., Tani H., Baba Y., Harashima H., Tokeshi M. (2015). A strategy for synthesis of lipid nanoparticles using microfluidic devices with a mixer structure. RSC Adv..

[231] Nguyen D.P., Kloosterman F., Barbieri R., Brown E.N., Wilson M.A., Klausberger T.Z. (2005). Micromixers—A review. J. Micromech. Microeng..

[232] Zimmermann T.S., Lee A.C., Akinc A., Bramlage B., Bumcrot D., Fedoruk M.N., Harborth J., Heyes J.A., Jeffs L.B., John M. (2006). RNAi-mediated gene silencing in non-human primates. Nature.

[233] Liu D., Zhang H., Fontana F., Hirvonen J.T., Santos H.A. (2018). Current developments and applications of microfluidic technology toward clinical translation of nanomedicines. Adv. Drug Deliv. Rev..

[234] Schikarski T., Trzenschiok H., Peukert W., Avila M. (2019). Inflow boundary conditions determine T-mixer efficiency. React. Chem. Eng..

[235] Günther A., Khan S.A., Thalmann M., Trachsel F., Jensen K.F. (2004). Transport and reaction in microscale segmented gas–liquid flow. Lab Chip.

[236] Tiboni M., Tiboni M., Pierro A., Del Papa M., Sparaventi S., Cespi M., Casettari L. (2021). Microfluidics for nanomedicines manufacturing: An affordable and low-cost 3D printing approach. Int. J. Pharm..

[237] Camarri S., Mariotti A., Galletti C., Brunazzi E., Mauri R., Salvetti M.V. (2020). An Overview of Flow Features and Mixing in Micro T and Arrow Mixers. Ind. Eng. Chem. Res..

[238] Jahn A., Stavis S.M., Hong J.S., Vreeland W.N., DeVoe D.L., Gaitan M. (2010). Microfluidic Mixing and the Formation of Nanoscale Lipid Vesicles. ACS Nano.

[239] Jahn A., Vreeland W.N., DeVoe D.L., Locascio L.E., Gaitan M. (2007). Microfluidic Directed Formation of Liposomes of Controlled Size. Langmuir.

[240] Capretto L., Carugo D., Mazzitelli S., Nastruzzi C., Zhang X. (2013). Microfluidic and lab-on-a-chip preparation routes for organic nanoparticles and vesicular systems for nanomedicine applications. Adv. Drug Deliv. Rev..

[241] Webb C., Khadke S., Tandrup Schmidt S., Roces C.B., Forbes N., Berrie G., Perrie Y. (2019). The Impact of Solvent Selection: Strategies to Guide the Manufacturing of Liposomes Using Microfluidics. Pharmaceutics.

[242] Krzysztoń R., Salem B., Lee D.J., Schwake G., Wagner E., Rädler J.O. (2017). Microfluidic self-assembly of folate-targeted monomolecular siRNA-lipid nanoparticles. Nanoscale.

[243] van Swaay D., DeMello A. (2013). Microfluidic methods for forming liposomes. Lab Chip.

[244] Mijajlovic M., Wright D., Zivkovic V., Bi J.X., Biggs M.J. (2013). Microfluidic hydrodynamic focusing based synthesis of POPC liposomes for model biological systems. Colloids Surf. B Biointerfaces.

[245] Balbino T.T.A., Aoki N.T., Gasperini A.A.M., Oliveira C.L.P., Azzoni A.R., Cavalcanti L.P., de la Torre L.G. (2013). Continuous flow production of cationic liposomes at high lipid concentration in microfluidic devices for gene delivery applications. Chem. Eng. J..

[246] Hood R.R., Shao C., Omiatek D.M., Vreeland W.N., DeVoe D.L. (2013). Microfluidic Synthesis of PEG- and Folate-Conjugated Liposomes for One-Step Formation of Targeted Stealth Nanocarriers. Pharm. Res..

[247] Church A.S., Witting M.D. (1997). Laboratory testing in ethanol, methanol, ethylene glycol, and isopropanol toxicities. J. Emerg. Med..

[248] Wu N., Zhu Y., Leech P., Sexton B., Brown S., Easton C. Effects of Surfactants on the Formation of Microdroplets in the Flow Focusing Microfluidic Device. Proceedings of the BioMEMS and Nanotechnology III.

[249] ICH (2021). Guideline Q3C (R8) on Impurities: Guideline for Residual Solvents. European Medicines Agency. EMA/CHMP/ICH/82260/2006..

[250] Joshi S., Hussain M.T., Roces C.B., Anderluzzi G., Kastner E., Salmaso S., Kirby D.J., Perrie Y. (2016). Microfluidics based manufacture of liposomes simultaneously entrapping hydrophilic and lipophilic drugs. Int. J. Pharm..

[251] Lou G., Anderluzzi G., Woods S., Roberts C.W., Perrie Y. (2019). A novel microfluidic-based approach to formulate size-tuneable large unilamellar cationic liposomes: Formulation, cellular uptake and biodistribution investigations. Eur. J. Pharm. Biopharm..

[252] Hood R.R., DeVoe D.L., Atencia J., Vreeland W.N., Omiatek D.M. (2014). A facile route to the synthesis of monodisperse nanoscale liposomes using 3D microfluidic hydrodynamic focusing in a concentric capillary array. Lab Chip.

[253] Allen T.M., Cullis P.R. (2013). Liposomal drug delivery systems: From concept to clinical applications. Adv. Drug Deliv. Rev..

[254] Chang H.I., Yeh M.K. (2012). Clinical development of liposome-based drugs: Formulation, characterization, and therapeutic efficacy. Int. J. Nanomed..

[255] Yang J.-T., Fang W.-F., Tung K.-Y. (2008). Fluids mixing in devices with connected-groove channels. Chem. Eng. Sci..

[256] van Schijndel T., Singh M.K., Gillies M., Kahya N., Kharin A., den Toonder J.M.J. (2011). Toward Gradient Formation in Microfluidic Devices by using Slanted Ridges. Macromol. Mater. Eng..

[257] Lin D., He F., Liao Y., Lin J., Liu C., Song J., Cheng Y. (2013). Three-dimensional staggered herringbone mixer fabricated by femtosecond laser direct writing. J. Opt..

[258] Chen D., Love K.T., Chen Y., Eltoukhy A.A., Kastrup C., Sahay G., Jeon A., Dong Y., Whitehead K.A., Anderson D.G. (2012). Rapid Discovery of Potent siRNA-Containing Lipid Nanoparticles Enabled by Controlled Microfluidic Formulation. J. Am. Chem. Soc..

[259] Ianovska M. (2018). Microfluidic Tools for Multidimensional Liquid Chromatography. Ph.D. Thesis.

[260] Kauffman K.J., Dorkin J.R., Yang J.H., Heartlein M.W., DeRosa F., Mir F.F., Fenton O.S., Anderson D.G. (2015). Optimization of Lipid Nanoparticle Formulations for mRNA Delivery in Vivo with Fractional Factorial and Definitive Screening Designs. Nano Lett..

[261] Gooding O.W. (2004). Process optimization using combinatorial design principles: Parallel synthesis and design of experiment methods. Curr. Opin. Chem. Biol..

[262] Dahlman J.E., Kauffman K.J., Xing Y., Shaw T.E., Mir F.F., Dlott C.C., Langer R., Anderson D.G., Wang E.T. (2017). Barcoded nanoparticles for high throughput in vivo discovery of targeted therapeutics. Proc. Natl. Acad. Sci. USA.

[263] Guimaraes P.P.G., Zhang R., Spektor R., Tan M., Chung A., Billingsley M.M., El-Mayta R., Riley R.S., Wang L., Wilson J.M. (2019). Ionizable lipid nanoparticles encapsulating barcoded mRNA for accelerated in vivo delivery screening. J. Control. Release.

[264] Kastner E., Verma V., Lowry D., Perrie Y. (2015). Microfluidic-controlled manufacture of liposomes for the solubilisation of a poorly water soluble drug. Int. J. Pharm..

[265] Hood R.R., DeVoe D.L. (2015). High-Throughput Continuous Flow Production of Nanoscale Liposomes by Microfluidic Vertical Flow Focusing. Small.

[266] Roces C.B., Lou G., Jain N., Abraham S., Thomas A., Halbert G.W., Perrie Y. (2020). Manufacturing Considerations for the Development of Lipid Nanoparticles Using Microfluidics. Pharmaceutics.

[267] Finn J.D., Smith A.R., Patel M.C., Shaw L., Youniss M.R., van Heteren J., Dirstine T., Ciullo C., Lescarbeau R., Seitzer J. (2018). A Single Administration of CRISPR/Cas9 Lipid Nanoparticles Achieves Robust and Persistent In Vivo Genome Editing. Cell Rep..

[268] Patel S., Ashwanikumar N., Robinson E., Xia Y., Mihai C., Griffith J.P., Hou S., Esposito A.A., Ketova T., Welsher K. (2020). Naturally-occurring cholesterol analogues in lipid nanoparticles induce polymorphic shape and enhance intracellular delivery of mRNA. Nat. Commun..

[269] Kimura N., Maeki M., Sato Y., Note Y., Ishida A., Tani H., Harashima H., Tokeshi M. (2018). Development of the iLiNP Device: Fine Tuning the Lipid Nanoparticle Size within 10 nm for Drug Delivery. ACS Omega.

[270] Balbino T.A., Azzoni A.R., de la Torre L.G. (2013). Microfluidic devices for continuous production of pDNA/cationic liposome complexes for gene delivery and vaccine therapy. Colloids Surf. B Biointerfaces.

[271] Kulkarni J.A., Chen S., Tam Y.Y.C. (2021). Scalable Production of Lipid Nanoparticles Containing Amphotericin B. Langmuir.

[272] Yang Z., Yu B., Zhu J., Huang X., Xie J., Xu S., Yang X., Wang X., Yung B.C., Lee L.J. (2014). A microfluidic method to synthesize transferrin-lipid nanoparticles loaded with siRNA LOR-1284 for therapy of acute myeloid leukemia. Nanoscale.

[273] Kim H., Sung J., Chang Y., Alfeche A., Leal C. (2018). Microfluidics Synthesis of Gene Silencing Cubosomes. ACS Nano.

[274] Sato Y., Note Y., Maeki M., Kaji N., Baba Y., Tokeshi M., Harashima H. (2016). Elucidation of the physicochemical properties and potency of siRNA-loaded small-sized lipid nanoparticles for siRNA delivery. J. Control. Release.

[275] Jyotsana N., Sharma A., Chaturvedi A., Budida R., Scherr M., Kuchenbauer F., Lindner R., Noyan F., Sühs K.-W., Stangel M. (2019). Lipid nanoparticle-mediated siRNA delivery for safe targeting of human CML in vivo. Ann. Hematol..

[276] Gkionis L., Campbell R.A., Aojula H., Harris L.K., Tirella A. (2020). Manufacturing drug co-loaded liposomal formulations targeting breast cancer: Influence of preparative method on liposomes characteristics and in vitro toxicity. Int. J. Pharm..

[277] Hamano N., Böttger R., Lee S.E., Yang Y., Kulkarni J.A., Ip S., Cullis P.R., Li S.-D. (2019). Robust Microfluidic Technology and New Lipid Composition for Fabrication of Curcumin-Loaded Liposomes: Effect on the Anticancer Activity and Safety of Cisplatin. Mol. Pharm..

[278] Fathordoobady F., Sannikova N., Guo Y., Singh A., Kitts D.D., Pratap-Singh A. (2021). Comparing microfluidics and ultrasonication as formulation methods for developing hempseed oil nanoemulsions for oral delivery applications. Sci. Rep..

[279] Dong Y.-D., Tchung E., Nowell C., Kaga S., Leong N., Mehta D., Kaminskas L.M., Boyd B.J. (2019). Microfluidic preparation of drug-loaded PEGylated liposomes, and the impact of liposome size on tumour retention and penetration. J. Liposome Res..

[280] Forbes N., Hussain M.T., Briuglia M.L., Edwards D.P., Horst J.H.t., Szita N., Perrie Y. (2019). Rapid and scale-independent microfluidic manufacture of liposomes entrapping protein incorporating in-line purification and at-line size monitoring. Int. J. Pharm..

[281] Khadke S., Roces C.B., Donaghey R., Giacobbo V., Su Y., Perrie Y. (2020). Scalable solvent-free production of liposomes. J. Pharm. Pharmacol..

[282] Salafi T., Zeming K.K., Zhang Y. (2017). Advancements in microfluidics for nanoparticle separation. Lab Chip.

[283] Petreus T., Cadogan E., Hughes G., Smith A., Pilla Reddy V., Lau A., O’Connor M.J., Critchlow S., Ashford M., Oplustil O’Connor L. (2021). Tumour-on-chip microfluidic platform for assessment of drug pharmacokinetics and treatment response. Commun. Biol..

[284] Sharma S., Bhatia V. (2021). Magnetic nanoparticles in microfluidics-based diagnostics: An appraisal. Nanomedicine.

[285] Lapierre F., Piret G., Drobecq H., Melnyk O., Coffinier Y., Thomy V., Boukherroub R. (2011). High sensitive matrix-free mass spectrometry analysis of peptides using silicon nanowires-based digital microfluidic device. Lab Chip.

[286] Ran R., Wang H.-F., Hou F., Liu Y., Hui Y., Petrovsky N., Zhang F., Zhao C.-X. (2019). A Microfluidic Tumor-on-a-Chip for Assessing Multifunctional Liposomes’ Tumor Targeting and Anticancer Efficacy. Adv. Healthc. Mater..

[287] Zizzari A., Bianco M., Carbone L., Perrone E., Amato F., Maruccio G., Rendina F., Arima V. (2017). Continuous-Flow Production of Injectable Liposomes via a Microfluidic Approach. Materials.

[288] Yanar F., Mosayyebi A., Nastruzzi C., Carugo D., Zhang X. (2020). Continuous-Flow Production of Liposomes with a Millireactor under Varying Fluidic Conditions. Pharmaceutics.

[289] Amador C., Gavriilidis A., Angeli P. (2004). Flow distribution in different microreactor scale-out geometries and the effect of manufacturing tolerances and channel blockage. Chem. Eng. J..

[290] Shah V.M., Nguyen D.X., Patel P., Cote B., Al-Fatease A., Pham Y., Huynh M.G., Woo Y., Alani A.W.G. (2019). Liposomes produced by microfluidics and extrusion: A comparison for scale-up purposes. Nanomed. Nanotechnol. Biol. Med..

[291] Roces C.B., Port E.C., Daskalakis N.N., Watts J.A., Aylott J.W., Halbert G.W., Perrie Y. (2020). Rapid scale-up and production of active-loaded PEGylated liposomes. Int. J. Pharm..

[292] Lamb Y.N. (2021). BNT162b2 mRNA COVID-19 Vaccine: First Approval. Drugs.

[293] Schoenmaker L., Witzigmann D., Kulkarni J.A., Verbeke R., Kersten G., Jiskoot W., Crommelin D.J.A. (2021). mRNA-lipid nanoparticle COVID-19 vaccines: Structure and stability. Int. J. Pharm..

[294] Roberts S.A., Parikh N., Blower R.J., Agrawal N. (2018). SPIN: Rapid synthesis, purification, and concentration of small drug-loaded liposomes. J. Liposome Res..

[295] Tenchov R., Bird R., Curtze A.E., Zhou Q. (2021). Lipid Nanoparticles─From Liposomes to mRNA Vaccine Delivery, a Landscape of Research Diversity and Advancement. ACS Nano.

[296] Stone N.R.H., Bicanic T., Salim R., Hope W. (2016). Liposomal Amphotericin B (AmBisome^®^): A Review of the Pharmacokinetics, Pharmacodynamics, Clinical Experience and Future Directions. Drugs.

[297] O’Brien M.E.R., Wigler N., Inbar M., Rosso R., Grischke E., Santoro A., Catane R., Kieback D.G., Tomczak P., Ackland S.P. (2004). Reduced cardiotoxicity and comparable efficacy in a phase IIItrial of pegylated liposomal doxorubicin HCl(CAELYX™/Doxil^®^) versus conventional doxorubicin forfirst-line treatment of metastatic breast cancer. Ann. Oncol..

[298] Zhang X., Goel V., Robbie G.J. (2020). Pharmacokinetics of Patisiran, the First Approved RNA Interference Therapy in Patients With Hereditary Transthyretin-Mediated Amyloidosis. J. Clin. Pharmacol..

[299] Matsuura-Sawada Y., Maeki M., Nishioka T., Niwa A., Yamauchi J., Mizoguchi M., Wada K., Tokeshi M. (2022). Microfluidic Device-Enabled Mass Production of Lipid-Based Nanoparticles for Applications in Nanomedicine and Cosmetics. ACS Appl. Nano Mater..

[300] Bresseleers J., Bagheri M., Lebleu C., Lecommandoux S., Sandre O., Pijpers I.A.B., Mason A.F., Meeuwissen S., Nostrum C.F.v., Hennink W.E. (2020). Tuning Size and Morphology of mPEG-b-p(HPMA-Bz) Copolymer Self-Assemblies Using Microfluidics. Polymers.

[301] Giraldo K.A., Bermudez J.S., Torres C.E., Reyes L.H., Osma J.F., Cruz J.C. (2021). Microfluidics for Multiphase Mixing and Liposomal Encapsulation of Nanobioconjugates: Passive vs. Acoustic Systems. Fluids.

[302] Rasouli M.R., Tabrizian M. (2019). An ultra-rapid acoustic micromixer for synthesis of organic nanoparticles. Lab Chip.

[303] Shepherd S.J., Warzecha C.C., Yadavali S., El-Mayta R., Alameh M.G., Wang L., Weissman D., Wilson J.M., Issadore D., Mitchell M.J. (2021). Scalable mRNA and siRNA Lipid Nanoparticle Production Using a Parallelized Microfluidic Device. Nano Lett..

[304] Rosenblum D., Gutkin A., Kedmi R., Ramishetti S., Veiga N., Jacobi A.M., Schubert M.S., Friedmann-Morvinski D., Cohen Z.R., Behlke M.A. (2020). CRISPR-Cas9 genome editing using targeted lipid nanoparticles for cancer therapy. Sci Adv..

[305] Riley R.S., Kashyap M.V., Billingsley M.M., White B., Alameh M.G., Bose S.K., Zoltick P.W., Li H., Zhang R., Cheng A.Y. (2021). Ionizable lipid nanoparticles for in utero mRNA delivery. Sci Adv..

[306] Sealy A. How Pfizer Makes Its Millions of COVID-19 Vaccine Doses. https://edition.cnn.com/2021/03/31/health/pfizer-vaccine-manufacturing/index.html.

