# Supplementary material for: Microfluidic Manufacture of Lipid-Based Nanomedicines
Source: Pharmaceutics. 2022 Sep 14;14(9):1940. doi: 10.3390/pharmaceutics14091940 (PMC9506151; doi:10.3390/pharmaceutics14091940)
Supplement: Supplementary file 1 [file pharmaceutics-14-01940-s001.zip › pharmaceutics-1878010-supplementary.pdf]

Supplementary files

Microfluidic Manufacture of Lipid-based Nanomedicines

Karim Osouli-Bostanabad [1,2], Sara Puliga [1], Dolores R. Serrano [3,4,*], Andrea Bucchi [5], Gavin Halbert [6] and Aikaterini Lalatsa [1,2,6,*]

1 Biomaterials, Bio-Engineering and Nanomedicine (BioN) Lab, Institute of Biomedical and Biomolecular, Sciences, School of Pharmacy and Biomedical Sciences, University of Portsmouth, White Swan Road, Portsmouth PO1 2DT, UK
2 School of Pharmacy and Biomedical Sciences, Robertson Wing, University of Strathclyde, 161, Cathedral Street, Glasgow G4 0RE, UK
3 Pharmaceutics and Food Technology Department, School of Pharmacy, Universidad Complutense de Madrid, Plaza Ramón y Cajal s/n, 28040 Madrid, Spain
4 Facultad de Farmacia, Instituto Universitario de Farmacia Industrial, Universidad Complutense de Madrid, 28040 Madrid, Spain
5 School of Mechanical and Design Engineering, Faculty of Technology, University of Portsmouth, Portsmouth PO1 3DJ, UK
6 CRUK Formulation Unit, School of Pharmacy and Biomedical Sciences, Robertson Wing, University of Strathclyde, 161, Cathedral Street, Glasgow G4 0RE, UK
* Correspondence: dr.serrano@farm.ucm.es (D.R.S.); aikaterini.lalatsa@strath.ac.uk (A.L.); Tel.: +44-141-548-2675 (A.L.)

Table S1: Lipid-based formulations in clinical trials

System	Drug/ Target	Product name	Indication	Clinical trials	Sponsor	Ref
Liposomes	Mitoxantrone	Liposomal Mitoxantrone (LEM)	Tumours	Phase 1 Completed (NCT00024492)	INSYS Therapeutics Inc.	[63]
	Belotecan, CKD-602	PEGylated liposomal formulation of CKD-602, S-CKD602	Advanced Malignancies	Phase 1 Completed (NCT00177281)	University of Pittsburgh, ALZA	[12]
	Topotecan	Liposomal Topotecan (TLI)	Ovarian Cancer, Small Cell Lung Cancer, Advanced Solid Tumours	Phase 1 Completed (NCT00765973)	Spectrum Pharmaceuticals Inc.	[64]
	Doxorubicin	Liposomal Doxorubicin, TLD-1	Advanced Solid Tumours	Phase 1 Recruiting (NCT03387917)	Swiss group for clinical cancer research	[65]
	Topotecan	Topotecan liposomal, INX-0076	Advanced Solid Tumours	Phase 1	Inex Pharmaceuticals Corporation	[12]

	Paclitaxel	Liposome	Metastatic Breast Cancer	Phase 1 Completed (NCT00080418) Phase 2 Completed (NCT01190982) (NCT00100139)	INSYS Therapeutics Inc.	[63, 12]
	Oxaliplatin	Liposomal Oxaliplatin MBP-426	Advanced or Metastatic Solid Tumours	Phase 1 Recruiting Completed (NCT00355888)	Mebiopharm Co., Ltd	[66]
	Oxaliplatin / Transferrin	Liposomal Oxaliplatin, MBP-426/Leucovorin/5-FU	Second line metastatic gastric, gastro-esophageal junction or oesophageal adenocarcinoma	Phase 2 (NCT00964080)	Mebiopharm Co., Ltd	[67]
	Doxorubicin / Antibody fragment; human epidermal growth factor receptor 2 (HER2)	PEGylated liposome modified with antibodies, MM-302	Breast Cancer	Phase 1/2 Terminated (NCT02213744)	Merrimack Pharmaceuticals	[68]
	Cisplatin	Targeted liposomal cisplatin, LiPlaCis	Advanced / Refractory Tumours, Metastatic Breast, skin and prostate cancers	Phase1/2 (NCT01861496)	Allarity Therapeutics	[12]
	Cisplatin	Pegylated cisplatin liposomes, SPI-77	Ovarian Cancer	Phase 2 Completed (NCT00004083)	NYU Langone Health	[63]
	Lurtotecan	Liposomal Lurtotecan, OSI-211	Recurrent Small Cell Lung Cancer	Phase 2 Completed (NCT00046787)	Astellas Pharma Inc, OSI Pharmaceuticals	[63, 12]
	Doxorubicin	Doxorubicin & Microwave induced Hyperthermia, ThermoDox	Hepatocellular Carcinoma	Phase 3 Completed (NCT02112656)	Celsion	[69]
	Methotrexate	MBP-Y003 Transferrin functionalised liposomes	Lymphoma	NA	Mebiopharm Co., Ltd.	[70]

	Docetaxel	MBP – Y004 Transferrin functionalised liposomes				
	Gemcitabine	MBP – Y005 Transferrin functionalised liposomes				
Lipid complexes	Oncogene MYC (dsRNA)	DCR-MYC	Hepatocellular Carcinoma, Solid tumours	Phase 1/2 Terminated (NCT02314052, NCT02110563)	Dicerna Pharmaceuticals, Inc.	[71]
	Polo-like kinase-1 (PLK1)	TKM-080301	Neuroendocrine Tumours, Adrenocortical Carcinoma, Advanced Hepatocellular	Phase 1/2 Completed (NCT02191878, NCT01262235)	Arbutus Biopharma Corporation	[71]
	EPH Receptor A2 (EphA2) siRNA	EphA2	Advanced or Recurrent Solid Tumours	Phase 1 Active (NCT01591356)	M.D. Anderson Cancer Center, National Cancer Institute (NCI)	[71]
	Hepatitis B surface antigen (HBsAg) ds siRNAs	ARB-001467	Chronic hepatitis B virus Infection	Phase 2 Completed (NCT02631096)	Arbutus Biopharma Corporation	[71]
	Apolipoprotein B (ApoB) siRNA	PRO-040201	Hypercholesterolemia	Phase 1 Terminated (NCT00927459)	Arbutus Biopharma Corporation	[71]
	Proprotein convertase subtilisin/kexin type 9 (PCSK9) siRNA	ALN-PCS02	Hypercholesterolemia, Elevated LDL-Cholesterol (LDL-C)	Phase 1 Completed (NCT01437059)	Alnylam Pharmaceuticals	[71]
	Heat shock protein (HSP47) siRNA	siRNAND-L02-s0201	Hepatic fibrosis	Phase 1 Completed (NCT02227459)	Bristol-Myers Squibb, Nitto Denko Corporation	[71]
	HBsAg siRNA	ARC-520	Chronic Hepatitis B Virus	Phase 1 Terminated (NCT00927459)	Arrowhead Pharmaceuticals, ICON Clinical Research	[72]
	HBsAg siRNA	DCR HBVS	Chronic Hepatitis B Virus	Phase 1 Active (NCT03772249)	Dicerna Pharmaceuticals, Inc.	[72]
	siRNA-kinesin spindle protein	ALN-VSP02	Solid tumours	Phase 1 Completed	Alnylam Pharmaceuticals	[73]

	(KSP), Vascular endothelial growth factor (VEGF)			(NCT01158079)		
	OX40L T cell, IL-23, and IL-36γ antigen	Lipid Nanoparticle encapsulated mRNA-2752	Advanced Malignancies	Phase 1/2 Recruiting (NCT03739931)	ModernaTX, Inc., AstraZeneca	[74]
	OX40L T cell	Lipid Nanoparticle mRNA-2416	Relapsed ovarian cancer, Refractory Solid Tumours, Lymphoma	Phase 1 Active (NCT03323398)	ModernaTX, Inc.	[75]
	Prokaryotic DNA repair enzyme	Liposomal T4N5 Lotion	Basal skin cell carcinoma, Actinic keratosis, Recurrent skin cancer, Squamous cell carcinoma	Phase 2 Completed (NCT00089180)	National Cancer Institute (NCI)	[76]
	$(CD)^{8+}$ and CD^{4+} T cell	Lipo-MERIT	Melanoma (Cancer)	Phase 1 Active (NCT02410733)	BioNTech SE	[77]
	Twenty tumour-associated antigens	mRNA-4157	Melanoma, Solid tumours	Phase 1 Active (NCT03313778, NCT03897881)	ModernaTX, Inc., Merck Sharp & Dohme Corp.	[78]
	MUC1	Liposomal L-BLP-25 (Stimuvax)	Non-Small Cell Lung Cancer, Multiple Myeloma	Phase 3 Terminated (NCT01015443)	Merck KGaA, Darmstadt, Germany	[63]
		BNT162b2	COVID-19	Phase 1/2, Phase 2/3 (NCT04380701, NCT04368728)	Pfizer-BioNTech	[79]
	S-2P antigen	mRNA-1273	Covid19, SARS-CoV2 Infection	Phase 1, 2, 3 (NCT04283461, NCT04405076,NCT04470427)	ModernaTX, Inc.	[80]

References:

12. Bulbake, U.; Doppalapudi, S.; Kommineni, N.; Khan, W. Liposomal Formulations in

Clinical Use: An Updated Review. *Pharmaceutics* **2017**, *9*, 12.
63. Fan, Y.; Marioli, M.; Zhang, K. Analytical characterization of liposomes and other lipid nanoparticles for drug delivery. *J. Pharm. Biomed. Anal.* **2021**, *192*, 113642.
64. Lamichhane, N.; Udayakumar, T.S.; D'Souza, W.D.; Simone II, C.B.; Raghavan, S.R.; Polf, J.; Mahmood, J. Liposomes: Clinical Applications and Potential for Image-Guided Drug Delivery. *Molecules* **2018**, *23*, 288.
65. Spectrum Pharmaceuticals, I. Topotecan Liposomes Injection for Small Cell Lung Cancer (SCLC), Ovarian Cancer and Other Advanced Solid Tumors. Availabe online: https://clinicaltrials.gov/ct2/show/NCT00765973 (accessed on 13 November 2020).
66. Research, S.G.f.C.C. TLD-1, a Novel Liposomal Doxorubicin, in Patients With Advanced Solid Tumors. Availibale online: https://clinicaltrials.gov/ct2/show/NCT03387917 (accessed on 7 September 2022).
67. Mebiopharm Co., L. Safety Study of MBP-426 (Liposomal Oxaliplatin Suspension for Injection) to Treat Advanced or Metastatic Solid Tumors. *clinicaltrials.gov* Availibale online: https://clinicaltrials.gov/ct2/show/NCT00355888 (accessed on 2 December 2014).
68. Mebiopharm Co., L. Study of MBP-426 in Patients With Second Line Gastric, Gastroesophageal, or Esophageal Adenocarcinoma. Availibale online: https://www.clinicaltrials.gov/ct2/show/NCT00964080 (accessed on 2 December 2014).
69. Munster, P.; Krop, I.E.; LoRusso, P.; Ma, C.; Siegel, B.A.; Shields, A.F.; Molnár, I.; Wickham, T.J.; Reynolds, J.; Campbell, K.; et al. Safety and pharmacokinetics of MM-302, a HER2-targeted antibody–liposomal doxorubicin conjugate, in patients with advanced HER2-positive breast cancer: a phase 1 dose-escalation study. *Br. J. Cancer* **2018**, *119*, 1086-1093. https://doi:10.1038/s41416-018-0235-2.
70. Celsion. Study of ThermoDox With Standardized Radiofrequency Ablation (RFA) for Treatment of Hepatocellular Carcinoma (HCC) (OPTIMA). Availibale online: https://clinicaltrials.gov/ct2/show/NCT02112656 (accessed on 24 October 2018).
71. Mebiopharm Co., L. Active targeting drug delivery system. Available online: http://www.mebiopharm.com/english/pro.html (accessed on 2021).
72. Yonezawa, S.; Koide, H.; Asai, T. Recent advances in siRNA delivery mediated by lipid-based nanoparticles. Adv. Drug Deliv. Rev. **2020**, *154-155*, 64-78. https://doi:https://doi.org/10.1016/j.addr.2020.07.022.
73. Ely, A.; Singh, P.; Smith, T.S.; Arbuthnot, P. In vitro transcribed mRNA for expression of designer nucleases: Advantages as a novel therapeutic for the management of chronic HBV infection. Adv. Drug Deliv. Rev. **2021**, *168*, 134-146. https://doi:https://doi.org/10.1016/j.addr.2020.05.010.
74. McGoron, A.J. Perspectives on the Future of Nanomedicine to Impact Patients: An Analysis of US Federal Funding and Interventional Clinical Trials. *Bioconjugate Chem.* **2020**, *31*, 436-447, https://doi:10.1021/acs.bioconjchem.9b00818.
75. ModernaTX, I., AstraZeneca. Dose Escalation Study of mRNA-2752 for Intratumoral Injection to Participants in Advanced Malignancies. Availibale online: https://clinicaltrials.gov/ct2/show/NCT03739931 (accessed on 18 July 2022).
76. ModernaTX, I. Dose Escalation and Efficacy Study of mRNA-2416 for Intratumoral Injection Alone and in Combination With Durvalumab for Participants With Advanced Malignancies. Availibale online: https://clinicaltrials.gov/ct2/show/NCT03323398 (accessed on 11 July 2022).
77. Institute, N.C. T4N5 Liposomal Lotion in Preventing The Recurrence of Nonmelanoma

Skin Cancer in Patients Who Have Undergone a Kidney Transplant. Availibale online: https://clinicaltrials.gov/ct2/show/NCT00089180 (accessed on 4 December 2015).
78. SE, B. Evaluation of the Safety and Tolerability of i.v. Administration of a Cancer Vaccine in Patients With Advanced Melanoma (Lipo-MERIT). Availibale online: https://clinicaltrials.gov/ct2/show/NCT02410733 (accessed on 24 August 2022).
79. ModernaTX, I., Merck Sharp & Dohme Corp. An Efficacy Study of Adjuvant Treatment With the Personalized Cancer Vaccine mRNA-4157 and Pembrolizumab in Participants With High-Risk Melanoma (KEYNOTE-942). Availibale online: https://clinicaltrials.gov/ct2/show/NCT03897881 (accessed on 24 August 2022).
80. Ebinger, J.E.; Fert-Bober, J.; Printsev, I.; Wu, M.; Sun, N.; Prostko, J.C.; Frias, E.C.; Stewart, J.L.; Van Eyk, J.E.; Braun, J.G.; et al. Antibody responses to the BNT162b2 mRNA vaccine in individuals previously infected with SARS-CoV-2. **Nat. Med. 2021**, *27*, 981-984. https://doi:10.1038/s41591-021-01325-6.
